# BOVIDS: A deep learning‐based software package for pose estimation to evaluate nightly behavior and its application to common elands (*Tragelaphus oryx*) in zoos

**DOI:** 10.1002/ece3.8701

**Published:** 2022-03-14

**Authors:** Jennifer Gübert, Max Hahn‐Klimroth, Paul W. Dierkes

**Affiliations:** ^1^ Faculty of Biological Sciences Bioscience Education and Zoo Biology Goethe University Frankfurt Germany; ^2^ Faculty of Computer Science TU Dortmund University Dortmund Germany

**Keywords:** deep learning, nightly behavior, posture estimation, REM sleep, *Tragelaphus oryx*, video action classification

## Abstract

Only a few studies on the nocturnal behavior of African ungulates exist so far, with mostly small sample sizes. For a comprehensive understanding of nocturnal behavior, the data basis needs to be expanded. Results obtained by observing zoo animals can provide clues for the study of wild animals and furthermore contribute to a better understanding of animal welfare and better husbandry conditions in zoos. The current contribution reduces the lack of data in two ways. First, we present a stand‐alone open‐source software package based on deep learning techniques, named *B*ehavioral *O*bservations by *V*ideos and *I*mages using *D*eep‐Learning *S*oftware (BOVIDS). It can be used to identify ungulates in their enclosure and to determine the three behavioral poses “Standing,” “Lying—head up,” and “Lying—head down” on 11,411 h of video material with an accuracy of 99.4%. Second, BOVIDS is used to conduct a case study on 25 common elands (*Tragelaphus oryx*) out of 5 EAZA zoos with a total of 822 nights, yielding the first detailed description of the nightly behavior of common elands. Our results indicate that age and sex are influencing factors on the nocturnal activity budget, the length of behavioral phases as well as the number of phases per behavioral state during the night while the keeping zoo has no significant influence. It is found that males spend more time in REM sleep posture than females while young animals spend more time in this position than adult ones. Finally, the results suggest a rhythm between the Standing and Lying phases among common elands that opens future research directions.

## Graphical abstract

The goal of this paper is two‐fold. First, a case study on 25 common elands (*Tragelaphus oryx*) out of 5 EAZA zoos with a total of 11,411 h of video material out of 822 nights is conducted, yielding the first detailed description of the nightly behavior of common elands. The second goal is the presentation of an open‐source software package named BOVIDS which is based on recent machine learning techniques and is designed to handle large‐scale studies of nocturnal ungulates’ behavior.

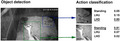

## 1 INTRODUCTION

### 1.1 General

The nocturnal behavior of many African mammals is poorly studied. It is known that the behavioral patterns can vary greatly between day and night, as many large herbivorous mammals spend, especially in winter, most of their sleeping time during the night, while the activity patterns emerge primarily at daytime (Bennie et al., 2014; Davimes et al., 2018; Gravett et al., 2017; Wu et al., 2018). For a comprehensive understanding of diurnal rhythms, a behavioral description of the entire diurnal cycle is necessary. Currently, there are only few contributions studying the nocturnal behavior. It is much more accessible to observe zoo animals at night rather than animals in their natural habitat due to much easier installation options of the required equipment (Ryder & Feistner, 1995). In order not to disturb the animals, camera recordings are a good mean of data collection in this case. Data collected in zoos can be valuable to study animal's behavior. In various species, there are no differences found in the behavior of animals in the wild and in captivity (Hollén & Manser, 2007; Melfi & Feistner, 2002). This was verified recently for basic nocturnal activities like being in the REM sleep position between giraffes in zoos and in the wild (Burger et al., 2020). Therefore, studies conducted in zoos can provide a good basis for describing the animals’ nocturnal behavior and the obtained results can subsequently serve as starting information for observations in the field (Burger et al., 2020). In addition, a deeper knowledge of nocturnal behavior inside zoo enclosures could contribute information to further improve animal management and husbandry in zoos (Brando & Buchanan‐Smith, 2018) and provide conclusions on animal welfare (Walsh et al., 2019). One explicit example is that REM sleep appears to be an important indicator of stress in giraffes (Sicks, 2016), which can be measured by noninvasive methods.

To describe nocturnal behavior unambiguously, reliable data are needed, especially because there are few comparisons in literature. This means that it would be preferable to observe multiple individuals of a species over a longer period of time to accurately describe the average behavior. Additionally, it is necessary to obtain data not only on one but on various species to close the existing knowledge gap. The extraction of meaningful information as well as a detailed evaluation of a mass of recorded data requires modern techniques to automate parts of this data mining process (Beery et al., 2020; Lürig et al., 2021; Norouzzadeh et al., 2018). In the last decade, various computer vision and deep learning techniques found their way into behavioral biology and ecology (Chakravarty et al., 2020; Dell et al., 2014; Eikelboom et al., 2019; Gerovichev et al., 2021; Norouzzadeh et al., 2021; Schneider et al., 2018, 2020; Valletta et al., 2017), facilitating the task of dealing with a large dataset. Unfortunately, automatization of the evaluation of video recordings is challenging if the video recordings suffer from a very low framerate (lower than 5 fps), much background noise, or heavy truncation effects, as is usual in observations in stables as zoo enclosures, or even in installments in the wild. More precisely, background noise appearing in such recordings is, for instance, due to light reflections caused by infrared emitters and particulate matter caused by the hay, while truncation and occlusion effects appear if the camera is not able to capture the whole enclosure or there are multiple overlapping animals in one stable. It is to emphasize that those negative effects are stronger the more general the setup is. Systems for automatic detection of flies or mice under perfect laboratory conditions (Graving et al., 2019; Kabra et al., 2013; Pereira et al., 2020) need to be much less robust to such effects than the system at hand for enclosures and stables. Of course, installments in the wild, like camera‐trap studies, must deal with even more noise and truncation.

### 1.2 Our contribution

One of the two main objectives of this work tackles this challenge by making BOVIDS (*B*ehavioral *O*bservations by *V*ideos and *I*mages using *D*eep‐Learning *S*oftware) available, which is a stand‐alone software package based on deep learning techniques. To the best of our knowledge, this is the first fully open‐source software package tackling the task of evaluating the nocturnal behavior of stalled animals that contains functionalities required for data preparation, training of the deep learning parts, data prediction, and data presentation. More precisely, BOVIDS can be used to evaluate video recordings of stalled ungulates recorded at 1 fps regarding two classification tasks: “binary classification” (a two‐class classification task) and “total classification” (a three‐class classification task), which are defined by Hahn‐Klimroth et al. (2021) as follows. First, if an animal is not present on an image, the desired label is Out (being out of view) in both tasks. Second, in the total classification task, the three postures Standing, Lying—head up (LHU), and Lying—head down (LHD) need to be distinguished which will be properly defined in Section 2.2. The binary classification task asks only to distinguish Standing and Lying (combining LHU and LHD) if the animal is present. All discussed software as well as detailed instructions can be found in our GitHub repository: https://github.com/Klimroth/BOVIDS and on Zenodo (https://doi.org/10.5281/zenodo.6143896).

As a second part of the paper, a case study is conducted that explains how BOVIDS can be applied by behavioral biologists to their own data and which statistical analyses can be directly conducted on the output of the software package. In this study, the nocturnal activity budget of 25 common elands is analyzed. To the best of our knowledge, the case study provides the first description of the nocturnal behavior of common elands. Over 11,000 h (822 nights) of video material from five different EAZA zoos were evaluated, a task that seems challenging in the absence of automatic evaluation and it is described in detail how BOVIDS can be used to observe and analyze several important behavioral biological key figures of nocturnal activity. The results contain activity budgets, which show the percentages of all examined behavioral states, a visualization of the Standing–Lying rhythm as well as an analysis of the possible influencing factors age, sex, and zoo husbandry.

### 1.3 Related work

As mentioned earlier, several computational systems have found their way into behavioral biology and ecology (Chakravarty et al., 2020; Dell et al., 2014; Eikelboom et al., 2019; Norouzzadeh et al., 2021; Valletta et al., 2017). Such systems are explicitly designed with respect to the underlying data. In the easiest tasks, cameras can be installed in a laboratory such that the recordings feature a high contrast between animals and the background as well as other laboratory conditions like a given steady camera angle and low background noise. Examples for such systems working with data of *Drosophila* flies or mice are *JAABA* (Kabra et al., 2013), *DeepBehavior* (Graving et al., 2019), and *SLEAP* (Pereira et al., 2020). When data are recorded either in the natural habitat or in different zoo enclosures, it is much more challenging to collect appropriate data that are amenable to automatic evaluation, for instance due to variations in weather, brightness, and background. Furthermore, different cameras can rarely be adjusted in a way such that the recording angle matches the given requirements or to ensure that animals are not highly truncated. It is to emphasize that there are examples of systems that deal with those challenges. One approach under varying brightness conditions distinguishes the poses “Lying” and “Standing” of cows in free‐stall stables (Porto et al., 2013). Furthermore, one success story is the work by Norouzzadeh et al. (2018, 2021) whose system can automatically detect and count different species, and some shown behaviors using camera trap images of the Serengeti dataset (Swanson et al., 2015). Similar systems working with camera trap images in the wild are presented by Schneider et al. (2020, 2018).

## 2 MATERIALS AND METHODS

As the purpose of this paper is two‐fold, this section is divided into several parts. In the section *Data evaluation*, methods and material used to collect the data of the case study and to evaluate the findings statistically are presented. Subsequently, the behavioral states of interest are defined properly in section *Ethogram*, whereas the section *Foundations of Deep Learning* introduces important concepts of machine learning used by BOVIDS. Finally, the section BOVIDS introduces and describes the single parts of the software package itself in more detail.

### 2.1 Data evaluation

The dataset includes nights of 25 common elands (*Tragelaphus oryx*), whereas the number of nights per individual ranges from 15 to 49. In total, 822 nights with 11,411 h of video material are present. The data were collected in winter seasons between 2017 and 2020 in a total of five EAZA zoos in Germany (Allwetterzoo Münster, Erlebnis‐Zoo Hannover, Opel‐Zoo Kronberg, Zoo Dortmund and Zoom Erlebniswelt Gelsenkirchen). A detailed overview about the used data is given in Table A1. For further analysis the individuals are categorized as follows: “young,” ranging from birth until the time of weaning with about six months, “subadult,” older than six months until sexual maturity with about two years of age and “adult” afterwards. These categories are chosen according to the information distributed across multiple prior works (Groves & Leslie, 2011; Myers et al., 2021; Puschmann et al., 2009; Tacutu et al., 2013).

All collected data are in the form of video recordings. The cameras used are capable of night vision due to built‐in infrared emitters (Lupus LE139HD or Lupus LE338HD with the recording device LUPUSTEC LE800HD or TECHNAXX PRO HD 720P). The recordings are made with a frame rate of 1 fps and the resolution ranges from 704 × 576 px to 1920 × 1080 px. Recording takes place in the stable during night, the time of the absence of animal keepers, which mostly ranges from 17:00 to 07:00 (14 h). In some cases, the recording time is 18:00 to 07:00 (13 h).

The data were recorded continuously providing an exact time span for every behavior with a start and an end time (Martin & Bateson, 2015). The manually annotation was governed by the open‐source program BORIS, Version 7.7.3 (Friard & Gamba, 2016) and consists of 2374 h of video material out of 170 nights. BOVIDS requires the use of multiple deep neural networks for object detection (OD) and action classification (AC) as explained by Hahn‐Klimroth et al. (2021) and in the following section. To train an initial object detection network, at least 400 images of every enclosure were annotated using LabelImg (Tzutalin, 2015) resulting in 11,326 images of common elands and 49,437 images of various African ungulates as already elaborated by Hahn‐Klimroth et al. (2021). Following the prescribed approach, the initial action classification networks were not only trained using 170 recordings (66,466 images) of common elands but also 113,407 images of other African ungulates with comparable postures. Furthermore, two rounds of offline hard example mining (OHEM) were conducted using additionally 14,381 images of common elands and 50,262 images of other African ungulates. Finally, the action classifiers used for common elands stalled together were fine‐tuned by 24,304 images stemming from manually annotated video files and 7377 images generated through OHEM. Detailed information can be found in Table A1.

All statistical analyses are conducted with R Studio (R Core Team, 2014) and the figures, which are not given by BOVIDS, are produced using the core functionalities of R and the package ggplot2 (Wickham, 2016). Statistical tests are performed differently for continuous and ordinal data. To conduct a two‐factor analysis of variance (ANOVA) on continuous data, normality is required which is tested by Shapiro–Wilk test for any behavior class. In case of significant deviation from normality (*p* < .05), a normality transformation is applied to the data by R’s “bestNormalize” package (Peterson & Cavanaugh, 2020). To analyze differences between multiple groups on ordinal data, a Kruskal‐Wallis test is applied. Finally, as post hoc tests on all pairs of potentially significant factors, a collection of unpaired t‐tests is applied in the continuous case and, respectively, a collection of Wilcoxon tests in the ordinal case. The alpha level is adjusted by the Bonferroni–Holm adjustment in each case.

### 2.2 Ethogram

**FIGURE 1:**
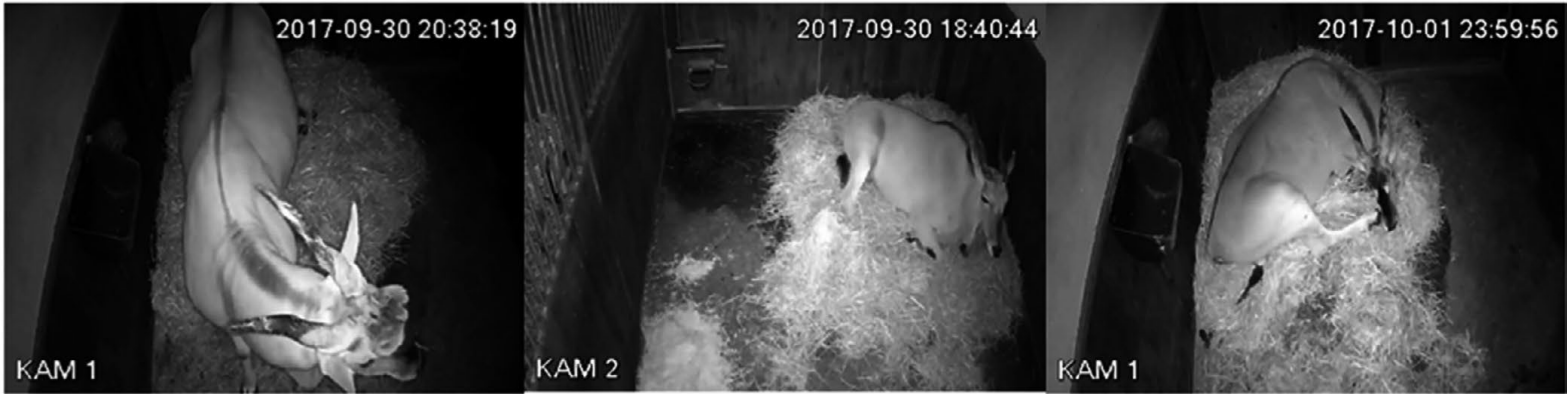
The three observed behavioral states: Standing, Lying—head up, Lying—head down, from left to right of common elands

The focus of this paper is to distinguish between three postures: Standing, Lying—head up (LHU), and Lying—head down (LHD). Finally, if there is no animal present, the assigned label is out of view (Out). The latter label can also be given if only a small part of the animal is visible, from which the posture cannot be inferred. Furthermore, the class Lying is defined as the union of LHU and LHD. The binary classification task which distinguishes Standing, Lying, and Out allows to analyze rhythms over the night as the categories “activity” and “rest” are the most prominently measured behavior stages to examine diurnal rhythms (Merrow et al., 2005). In the following ethogram, based on that of Hahn‐Klimroth et al. (2021), the three behavioral states are defined and shown in Figure 1.

Standing: The animal stands in an upright position on all four hooves. The exact behavior is neglected, thus the animal could be, for instance, feeding, resting, or ruminating.
Lying—head up (LHU): The animal lies down, and its head is lifted. The behavioral state does not distinguish if the animal is awake or in non‐REM sleep. As before, the precise behavior is neglected.
Lying—head down (LHD): The animal is lying with its head resting on the ground. The head's position is beside the body or sometimes in front of it.

It is crucial to notice that LHD is the typical REM (rapid eye movement) sleep posture. REM sleep is recognized through various behavioral components as the animal is lying with its head resting due to postural atonia (Lima et al., 2005; Zepelin et al., 2005). This characteristically REM sleep position can be used to estimate the REM sleep, a common approach in the study of behavior of common elands (Zizkova et al., 2013) and cows (Ternman et al., 2014).

### 2.3 Foundations of Deep Learning

In supervised machine learning tasks, one is usually interested to design a system that allows automatic prediction of new data based on manually annotated examples (Russell & Norvig, 2016). In this contribution, two excessively studied supervised learning tasks are employed: object detection and action classification.

In the easiest variant of the object detection task, an image is given as an input and the system is asked to draw a bounding box around the objects appearing in the image (bounding box regression) and to assign a class label that describes the content of each bounding box (classification). On a very high‐level description, there are two different approaches to this task. In one‐step object‐detection a bounding box is drawn, and the corresponding label is assigned simultaneously while in two‐step object‐detection, those tasks are conducted sequentially (Jiao et al., 2019). Well‐known representatives of one‐step solutions are yolo and SSD while there are various well‐known two‐step architectures like FasterRCNN, MaskRCNN, or EfficientDet. Without going into much detail, comparably modern one‐step architectures are mostly faster at the task as two‐step architectures but perform slightly worse in the classification part (Ouchra & Belangour, 2021).

Similarly, there is a huge set of deep learning architectures designed for the action classification task. In the easiest variant, an image is given, and the system needs to assign one unique class label out of a given set of labels (Lu & Weng, 2007). Prominent architectures are ResNet (He et al., 2016), EfficientNet (Tan & Le, 2019), or CoAtNet (Dai et al., 2021). The performance of such a classifier is measured by two important metrics: the accuracy as well as the f‐score (Tharwat, 2021).

Suppose a sequence of *n* images is predicted and image *i* gets label *s_i_* assigned by the classifier while its correct label, called ground‐truth, was *t_i_*. Suppose furthermore that classes 0, 1, …, *k* exist. Therefore, there are two sequences of labels $s={\left(t_{i}\right)}_{i=1\ldots n},t={\left(t_{i}\right)}_{i=1\ldots n}\in {\left\{0,\ldots ,k\right\}}^{n}$ which represent the classification by the neural network and the ground‐truth, respectively.

The accuracy is defined as the proportion of correctly labeled images among all images, or formally,

$$\text{accuracy}\left(s,t\right)=\frac{\left|\left\{i:s_{i}=t_{i}\right\}\right|}{n}.$$

The accuracy is a good indicator on how well a model performs on average, but if there are some underrepresented classes, the model's performance on those classes is not properly described by the accuracy. The f‐score, the harmonic mean of precision and recall, is a measure that describes the performance of a model per class quite well. To this end, let $tp\left(c,s,t\right)=\left|\left\{i:s_{i}=t_{i}=c\right\}\right|$ be the number of true positives classified by the model of class *c* and $fp\left(c,s,t\right)=\left|\left\{i:s_{i}=c,t_{i}\neq c\right\}\right|$ be the number of false positives, respectively. Analogously, define $fn\left(c,s,t\right)=\left|\left\{i:s_{i}\neq c,t_{i}=c\right\}\right|$ as the number of false negatives of class *c*. Then, the *f*‐score of class *c* can be expressed as

$$f\;\text{- score}\left(c,s,t\right)=\frac{tp\left(c,s,t\right)}{tp\left(c,s,t\right)+0.5\cdot \left(fp\left(c,s,t\right)+fn\left(c,s,t\right)\right)}.$$

While accuracy and f‐score capture important aspects of a deep learning model, only optimizing with respect to those metrics might not be sufficient in certain applications. Video action classification is such an example. Given a video file, the task is to train a model that can accurately predict the observed action at each time‐step of the video file. Very short misclassified sequences in a long video are clearly not captured by the f‐score or the accuracy but it causes classification flickering which might be problematic if one is interested in key quantities like the average length of certain activities. There are various recent developments in video action classification, most building up on so‐called “recurrent neural networks,” which have in common that multiple dimensions of the data given in the videos are used (Xu et al., 2016). First, there is a spatial dimension which is the evaluation of a single frame of the video file by classical action classification. Second, there is a temporal dimension given as the single frames are coming as a sequence and the evolution over time contains information. Capturing the temporal dimension with state‐of‐the‐art approaches becomes hard if the framerate of the video is very low (See & Rahman, 2015). A more classical approach toward employing the temporal dimension is the “multiple‐frame encoding” (Franche & Coulombe, 2012; Ji et al., 2013) in which subsequent frames are merged into one image that is fed into the model. This approach allows capturing the temporal dimension even given a low framerate, but it is inferior to more involved strategies as soon as the framerate increases (Xu et al., 2016). This multiple‐frame encoding will also be used in the present contribution, as the available video material is recorded with 1 frame per second.

In supervised learning tasks, a user presents the model a set of examples and the model is built upon those examples. This procedure is called training. More precisely, it is usual to split this set of examples into two parts: a training set and a validation set. During training, the accuracies of the model with respect to the training set as well as to the validation set are constantly measured and the model is optimized regarding the performance on the training set. In the survey by Wang et al. (2020), different metrics as the accuracy as target functions of this optimization process are discussed. While the performance on the training and validation data is of great theoretical interest, in applications, one is interested in the so‐called generalization accuracy. To measure this accuracy, a third dataset of manually annotated data points is required, the test set. The important difference between training and validation set is that the images in the test set were not presented to the model during training and, therefore, the model's performance on these data is a good indicator on how well the model will perform in an application. It is well‐known that the performance on the test set is better, the more similar the testing images are to the images presented during training. The discrepancy between the distribution of training images and testing images is called distribution shift and machine learning models are known to be brittle even to small distribution shifts (Quiñonero‐Candela et al., 2008) and, therefore, one tries to find a set of training images that represents the images in the application as best as possible.

### 2.4 BOVIDS

**FIGURE 2:**
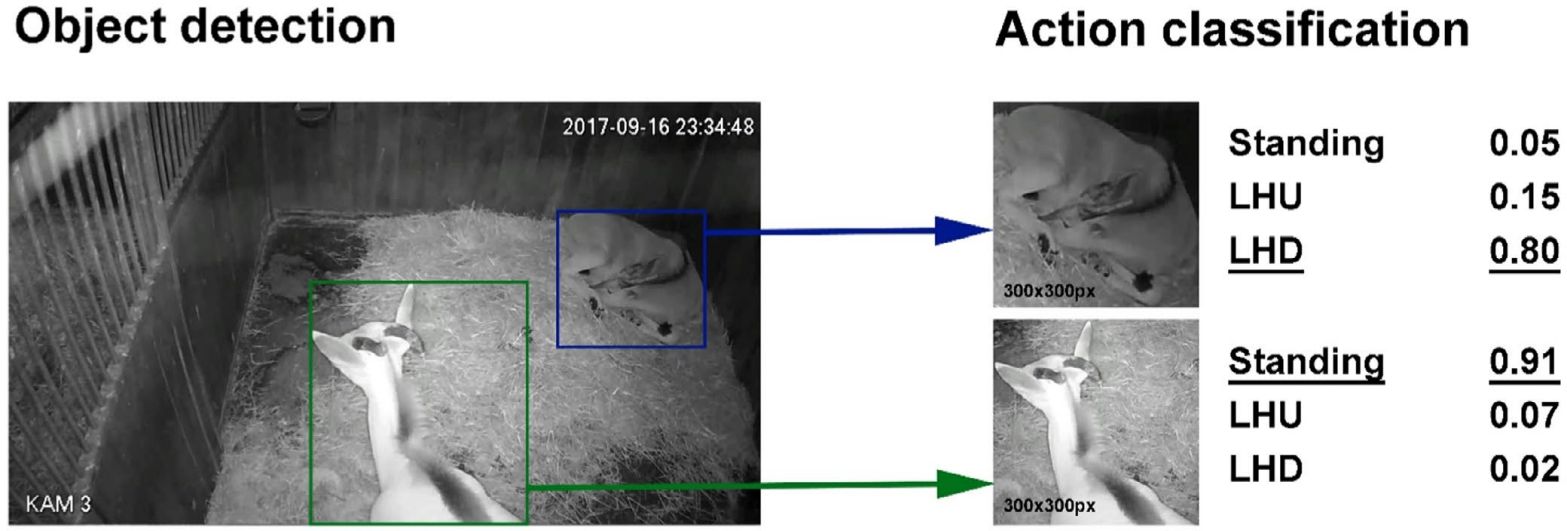
Visualization of the sequential application of the yolov4 object detector and the EfficientNet‐B3 action classifier

BOVIDS is an end‐to‐end software package which automatically identifies individuals of ungulates and their postures in videos. The detection itself is based on a sequential application of object detection and video action classification governed by state‐of‐the‐art deep neural networks, yolov4 (Bochkovskiy et al., 2020), and EfficientNet‐B3 (Tan & Le, 2019), see Figure 2. As explained, there are two classification tasks (total classification and binary classification). The object detector is used uniformly for both tasks while different sets of action classifiers are trained for either recognition of three classes or two classes, respectively.

It is important to emphasize that the following description is meant to present one possible way of using a deep‐learning pipeline, starting from data preparation, over training and evaluation, and ending with the preparation of real data for statistical analyses. Hereby, the used deep learning models perform well on testing sets and are known to be fast (Bochkovskiy et al., 2020; Tan & Le, 2019). The description is not meant to be the single possible way of implementing such a system. As will be shown, the system is easy to apply, and the results are satisfactory from a biologist's point of view.

#### 2.4.1 Overview

This section is devoted to give a short overview about BOVIDS’ functionality. The system is designed to achieve a good performance in long‐term studies using video recordings in enclosures. This includes observation of zoo animals as well as farm animal husbandry. The goal is to tell the posture (Standing, LHU, LHD) of the observed animals at any time in the video with high precision to describe its fundamental behavior as well as possible.

Manual annotation of a video file of one night (14 h) by a trained person requires roughly about two hours which indicates that only a few video files out of a longer observation period can be evaluated manually. This is a challenge as one is confronted with two problems in designing a valid training set for a deep learning model. First, the postures Standing, LHU, and LHD are highly imbalanced such that out of 14 h of video material, only a small portion can be easily used in a training set. It is of course possible to train on imbalanced data, but even this has limitations (Liu et al., 2019). Second, on different nights, the video recordings may vary due to changes in external conditions, like brightness or positioning of hay. Therefore, data recorded on different nights undergo a mild distribution shift. As manual annotation of many nights is very time‐consuming and annotation of random periods of each night might cause an even more severe class imbalance, this contribution suggests an adaptation of a process called “offline hard example mining” (Felzenszwalb et al., 2010). This approach tries to minimize human working load by the cost of higher computational cost in an iterative process. Miao et al. (2021) conducted an extensive study on such iterative processes and analyzed its performance with respect to deep‐learning models that evaluate camera‐trap images.

In the following section, a high‐level sketch of the functionalities of BOVIDS is given and the details can be found in the subsequent sections. BOVIDS is divided into four components:

BOV 1. Data collection,
BOV 2. Object detection (OD),
BOV 3. Action classification (AC),
BOV 4. Data prediction.

While a part of BOV 4 is a significantly improved and extended version of work presented in an earlier contribution (Hahn‐Klimroth et al., 2021), the newly developed components BOV 1–BOV 3 allow an interested user to apply the complete system comfortably to their own data. The software package consists of various small python scripts that allow to handle large datasets more conveniently and prepare the data in a way that can be used to apply the prediction pipeline BOV 4.

**FIGURE 3:**
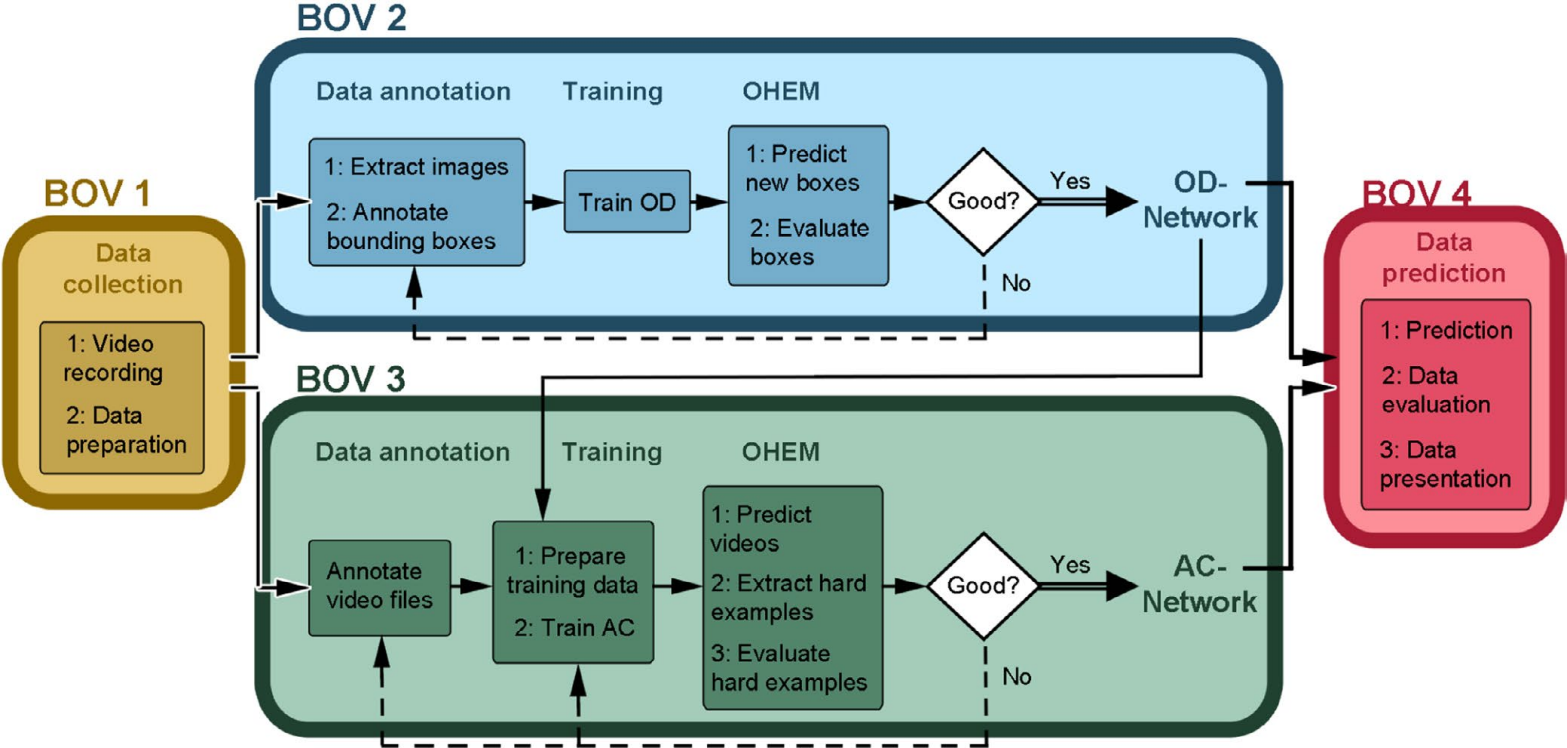
Overview of the System BOVIDS and all its categories

BOV 1 allows to convert video recordings directly from the LUPUS observation system. To annotate new data automatically, the prediction pipeline of BOVIDS (BOV 4) is used. The necessary scripts to prepare the training and validation set and to conduct the training are presented in BOV 2 for the object detector, while BOV 3 provides these functionalities with regard to the action classifier. Furthermore, those sections contain a description of one possibility to fine‐tune the models and achieve a good performance. Finally, multiple tools to measure the accuracy of the prediction and to detect systematic errors by BOVIDS are provided in BOV 4. Also, tools to represent and visualize the data that are a good starting point to apply further statistical methods are presented in this section. A visualization of the complete process is given in Figure 3.

#### 2.4.2 BOV 1: Data preparation

BOVIDS creates a collection of video files, one per night automatically if the data are recorded by the LUPUS observation system. If some data are missing due to power failure, the missing frames can be filled with a sequence of black frames to ensure a joint observation time over all video files. Such sequences of black frames will be labeled as Out by BOVIDS during prediction and, therefore, represent reality quite well.

#### 2.4.3 BOV 2: Training an object detector (OD)

The final object detector is trained following the subsequent procedure:

OD 1. Manual annotation of images.
OD 2. Train a first version of the object detector.
OD 3. Offline hard example mining (OHEM).
  a. Automatic annotation of unseen data.
  b. Evaluation of the suggested bounding boxes.
  c. Retrain the deep neural network.

In the initial annotation task (OD 1), between 400 and 800 images are sampled stemming from multiple videos per enclosure over the observation period to increase the data variability. The number of images sampled in total depends on how much data there are overall, how difficult the detection appears to be, and whether individuals need to be distinguished. Those images are annotated manually by a freely available third‐party software package called LabelImg (Tzutalin, 2015) and the initial training can be performed (OD 2). Hereby, 5% of the data is used as the validation set while 95% of the data is used for training.

**FIGURE 4:**
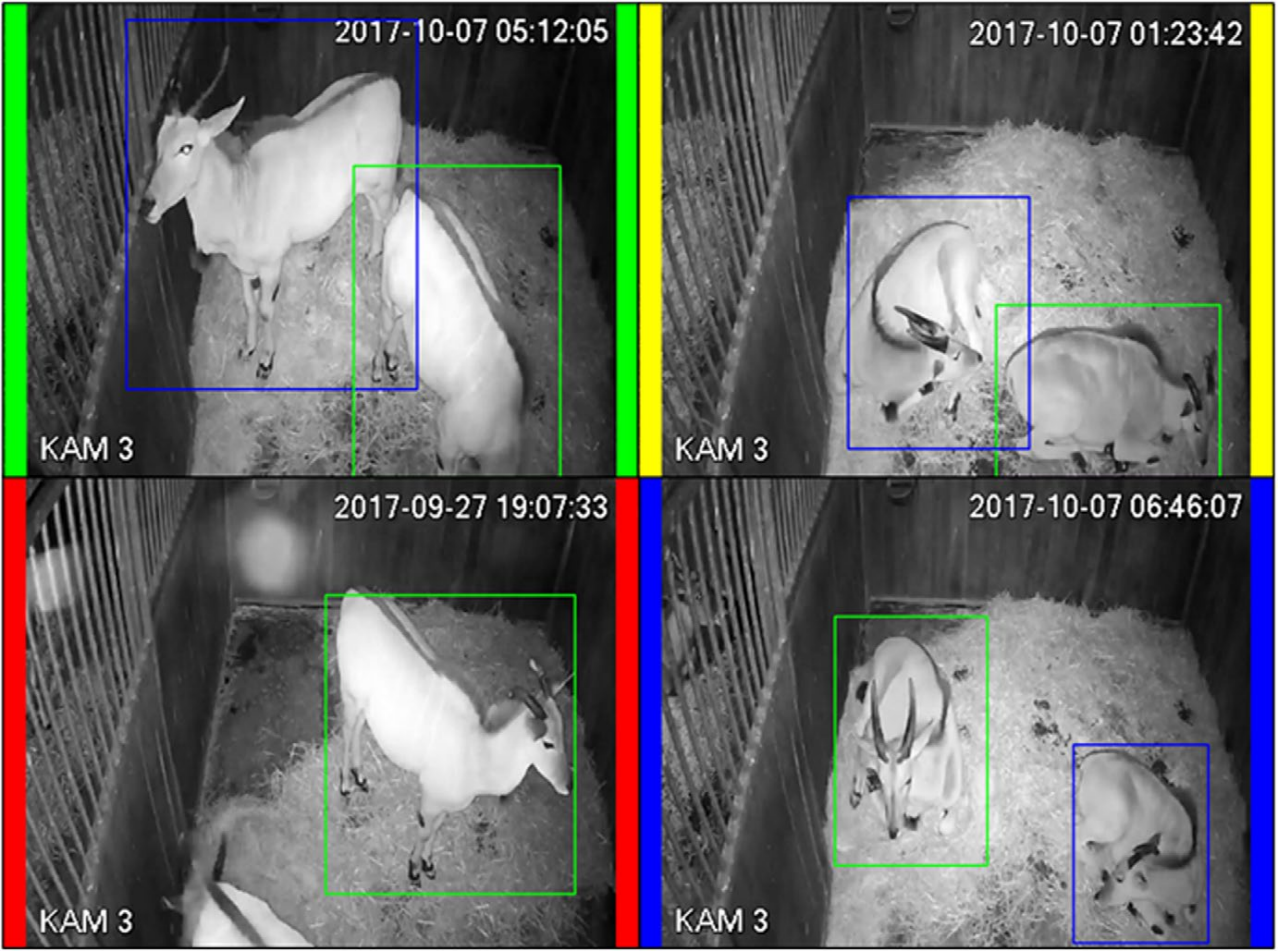
Example of the four classes that can be given in evaluation, good (green), okay (yellow), bad (red), and swapped (blue)

To run an adapted version of the so‐called “offline hard example mining” (Felzenszwalb et al., 2010), in short OHEM (OD 3), the object detector is used to automatically annotate 300–600 images out of unseen videos of the same set of enclosures (OD 3a). The quality of each such automatically drawn bounding box is evaluated. Hereby, a human assigns one out of four classes (good, okay, poor, swapped) to each bounding box (OD 3b) which is visualized in Figure 4. If the bounding boxes are satisfyingly accurate, the procedure stops at this point. Otherwise, the bounding boxes evaluated as poor or swapped are corrected manually using LabelImg. Those bounding boxes can be seen as “hard examples” as the current version of the object detector struggles at prediction. The freshly corrected annotations together with the well‐evaluated bounding boxes are used to increase the training set of the object detector and the object detector is trained on this new, extended set. Again, 5% of the existing data is used for validation. This procedure can be repeated until satisfying results are achieved. In the conducted case study, one iteration sufficed to achieve a decent accuracy. After having trained an accurately working object detector, this object detector is one ingredient required to generate a training set for the action classifiers.

#### 2.4.4 BOV 3: Action classification (AC)

The action classifier's goal is to predict the pose of an individual on a single image (single‐frame, SF) to capture the spatial dimension of the video, respectively, on four subsequent images placed next to each other (multiple‐frame, MF) to capture the temporal dimension. The case study suggests that the following iterative process generates a well‐performing action classifier and finds a good balance between accuracy of the deep learning model and human annotation time.

AC 1. Annotation of few video files.
AC 2. Training of a first version of the ACs.
  a. Preparation of an initial training set.
  b. Training of the ACs.
AC 3. One or multiple rounds of OHEM
  a. Prediction of many new video files.
  b. Extracting hard as well as random examples as single images.
  c. Manually evaluating the performance on those examples.
  d. Retrain the network based on the evaluated images.

When starting from scratch, it is most convenient to annotate the behavior of each single frame of a video by annotating the whole video (AC 1), for instance using the third‐party software package BORIS (Friard & Gamba, 2016). In the conducted case study, video material corresponding to 170 nights was annotated manually, see Table A1. To generate the training set, equally many images (single‐frame and multiple‐frame encoded) of each posture (Standing, LHU and LHD) are extracted from the annotated video files by using the previously trained object detector. This balancing is one possible way to ensure that training of the action classifiers works decently (Japkowicz & Stephen, 2002). The reader should be aware that there are different strategies to deal with class imbalance that will not be discussed in this contribution (Liu et al., 2019). Due to the class balancing and the underrepresentation of LHD in the video data, it is possible to extract roughly 500 images per class and per 14‐hour video on our dataset. To start training, a training set with 90% of those images and a validation set with 10% of those images are created (AC 2a). Finally, four EfficientNet‐B3 CNNs, namely the single‐frame classifier and the multiple‐frame classifier for both (binary and total) classification tasks (AC 2b) are trained.

These first versions of the action classifiers are supposed to work quite decently on the videos used for the training, but it is likely that the classification accuracy is worse on different videos of the same animal in which the arrangement of the enclosure as well as the light conditions might be quite different due to the already discussed distribution shift (Quiñonero‐Candela et al., 2008). For this reason, it seems sensible to reduce the distribution shift between the training set and the data required to be predicted by increasing the variability of the training data. To this end, we adapt the classical offline hard example mining to the setting at hand (AC 3) as follows. First, a fairly large number of momentarily not annotated video files will be predicted by BOVIDS (AC 3a). The accuracy of this prediction is expected to be at least 90% as Hahn‐Klimroth et al. (2021) already discussed. Therefore, BOVIDS provides an educated guess on the labels of each time interval of many video files that could not have been annotated manually without spending too much human annotation time. Based on those predicted labels, one samples a decent number of images in almost balanced classes distributed over the whole observation time (AC 3b). In the conducted case study, 72,020 images were sampled that way. These images are close to a uniform sample of the data on balanced classes of the whole underlying data and can, therefore, be referred to as “random” examples. These examples can now be evaluated by a human observer in a moderate amount of time (AC 3c). It is to be stressed at this point that a decent classifier is a critical ingredient: As the classes are highly unbalanced, random sampling without an educated guess would result in a set of images with almost no LHD, therefore, this simple process would not be possible to be used for generating a balanced training set.

Besides mining such random examples, it is also possible to extract “hard” examples easily. In this contribution, a hard example is defined as an image for which either the certainty of classification by the action classifier is small or if it belongs to a time interval of which the predictions of the single‐frame and multiple‐frame action classifier disagree. It is supposed that neural networks can be finetuned efficiently by hard examples (Felzenszwalb et al., 2010). Therefore, instead of only generating random samples distributed across the observation time, it is possible to nudge the training set into a direction such that information from momentarily hard to classify data gets boosted.

Based on the human evaluation of the single images it is now possible to retrain the action classifiers on a much broader dataset that really represents the distribution of the data that needs to be classified. At this point, the training classes might get slightly unbalanced if the human annotation deviates strongly from the automatic one. In this case standard techniques like random upsampling might be considered (Branco et al., 2016) and are provided by BOVIDS, of course, different ways to deal with this imbalance can also be employed (Liu et al., 2019). Once a decent object detector and a well‐performing action classifier are generated, all data can be predicted once more and the performance of BOVIDS can be measured.

At this point, we want to emphasize that training and validation data are generated as usual in machine learning for the object detection and the action classification tasks. However, generation of a suitable testing set and choosing a decent evaluation metric is more involved as the performances of the object detector and the action classifiers as single systems are subordinate to the outcome of their sequential application. This will be discussed in detail in Section 2.4.5.2.

#### 2.4.5 BOV 4: Data prediction

The data prediction step consists of three major parts (DP 1–DP 3) that are discussed in this section and are read as:

DP 1: Prediction
  P 1. Object detection phase
  P 2. Action classification phase
  P 3. Postprocessing phase.
DP 2: Data evaluation
DP 3: Data presentation.

##### DP 1: The prediction pipeline

The system of Hahn‐Klimroth et al. (2021) predicts a video file in three phases:

P 1. Object detection phase
P 2. Action classification phase
P 3. Postprocessing phase.

These phases must not be confused with BOV 2 and BOV 3 that describe how to train the required deep neural networks while P 1–P 3 are phases within the prediction pipeline of Hahn‐Klimroth et al. (2021) that require the previously trained networks. These phases are briefly explained below, and improvements and new features provided by BOVIDS, in contrast to the original system, are highlighted.

In the object detection phase (P 1), the system will first decompose a video file into so‐called “time intervals”. This is a discretization of the continuous data into packages of seven seconds each. More precisely, for each time interval the prediction pipeline will collect four images. Then, the object detector is used to identify the animal present in the images or, respectively, declare that no animal is present. While this step is governed by a Mask‐RCNN network by Hahn‐Klimroth et al. (2021) in the current version the architecture is changed to the much more recent yolov4 network as implemented by Taipingeric (2020) which improves the classification accuracy (Bochkovskiy et al., 2020) and significantly speeds up the complete prediction pipeline by approximately 40% on the same hardware. The merit of this step is two‐fold. First, as pointed out by Yosinski et al. (2014), it increases the similarity between images taken from different enclosures. This dramatically improves the chance of meaningful learning of the poses from various videos. Second, it is used to distinguish between distinct individuals within the same enclosure. At the end of the object detection phase, each time interval is represented in two ways for every individual: As a sequence of single images (single‐frame) and additionally as one image in which these images are placed next to each other (multiple‐frame encoded representation (Franche & Coulombe, 2012; Ji et al., 2013)).

The subsequent step, the action classification phase (P 2) to determine the behavioral classes, is a classical image classification task. For both, the single‐ and multiple‐frame representations, this task is governed by two independently trained EfficientNetB3 CNNs per time interval. The final prediction for any time interval is calculated as the average over both outcomes. Hahn‐Klimroth et al. (2021) already describe that the “total classification” task (distinguishing Standing, LHU, LHD) might be much more difficult than the “binary classification” task (distinguishing Standing and Lying) and gives the possibility to map the final prediction from LHU and LHD to Lying. The approach of BOVIDS toward this binary task is slightly different. It is necessary to train a set of independent networks that purely govern this binary classification such that possible features can eventually be better learned.

To reduce classification flickering, Hahn‐Klimroth et al. (2021) propose a set of postprocessing rules (P 3) which are applied to the sequence of classifications of time intervals. Those postprocessing rules dismiss very short sequences of a specific action as those sequences are likely to stem from short periods of false classifications. In the current setting the set of postprocessing rules is extended. It is now possible to handle flickering between Out and a specific behavior more smoothly to incorporate short periods in which the object detector failed to detect or identify the present individual. Of course, such a postprocessing step might dismiss short phases which are present in the data. Therefore, choosing an appropriate set of rules is a trade‐off between a stronger methodological error (errors made by BOVIDS through misclassification of short events) and a systematic error (errors caused by dismissing short phases on real data). BOVIDS contains tools for a systematic study of both types of errors. If the systematic error is appropriate for the application, one can compare BOVIDS’ prediction with the postprocessed real data to describe the methodological error.

In the present work, the chosen set of postprocessing rules varies significantly between the binary and the total classification task. Indeed, as the binary classification task is meant to study longer periods of Standing and Lying, phases up to 5 min are dismissed. Furthermore, in the total classification task, it is distinguished between adult common elands and nonadult common elands as the latter show shorter phases than the adult individuals. A detailed overview over the used postprocessing rules can be found in Table A2.

##### DP 2: Data evaluation

As the prediction of a deep learning‐based system works, in the end, as a black box, it is very important to assure the quality of the prediction regarding all quantities of interest. Therefore, it is crucial to define a valid testing set and appropriate evaluation metrics. Due to the iterative process on how the training set was found, the images used for training the action classifiers are an almost uniform sample from the whole observation period. Thus, any specific video is an adequate sample to determine the expected accuracy which implies that a good testing set is given by the already manually annotated videos. Observe that during training only the object detector and the action classifiers as single systems were evaluated with respect to a validation set but ultimately, it is more important that the prediction of a complete video is accurate with respect to biologically interesting quantities.

To quantify the accuracy of the prediction on the testing set, performance indicators from machine learning theory as well as biological key figures are evaluated by the following four quality criteria.

QC 1. Analysis of the object detector per night (“detection density”).
QC 2. Accuracy and f‐score as well as a comparison of the number of phases, the median phase length, and the overall percentage per activity class between BOVIDS’ prediction and the manual annotation.
QC 3. Number, length, and type of misclassified sequences.
QC 4. Visual checking for outliers.

While QC 2 and QC 3 are quality criteria which can be only evaluated with respect to the testing set, QC 1 and QC 4 can be applied to all predicted data.

In the first step (QC 1), the performance of the object detector should be checked in detail. It may happen that the object detector fails to detect the individual in certain videos quite often, which could be due to different light conditions or maybe because of heavy truncation. Of course, it is also possible that the individuals are Out for a longer period. To check the performance, BOVIDS outputs an overview that reports the percentage of detections of an individual by the object detector per video. If this value turns out to be noticeably low, one should check the original data to see if this low “detection density” can be explained.

Second, if the object detector works satisfactorily well and a good set of postprocessing rules was defined, the performance of the classification part of BOVIDS needs to be analyzed. Accuracy and f‐score (QC 2) are standard tools to measure the performance of a deep learning system. Those metrics are applied with respect to the postprocessed data in comparison to the manually annotated data to which the postprocessing rules were also applied. Further highly relevant biological quantities are the percentage per behavioral class and the median phase length where the latter is not evaluated appropriately by accuracy and f‐score. Finally, it is important to understand which kind of misclassifications occur and to, potentially, derive patterns. To analyze QC 2 and QC 3, BOVIDS contains a tool that allows to report the accuracy, f‐score, deviation in the number of phases as well as a detailed description of misclassified sequences.

**FIGURE 5:**
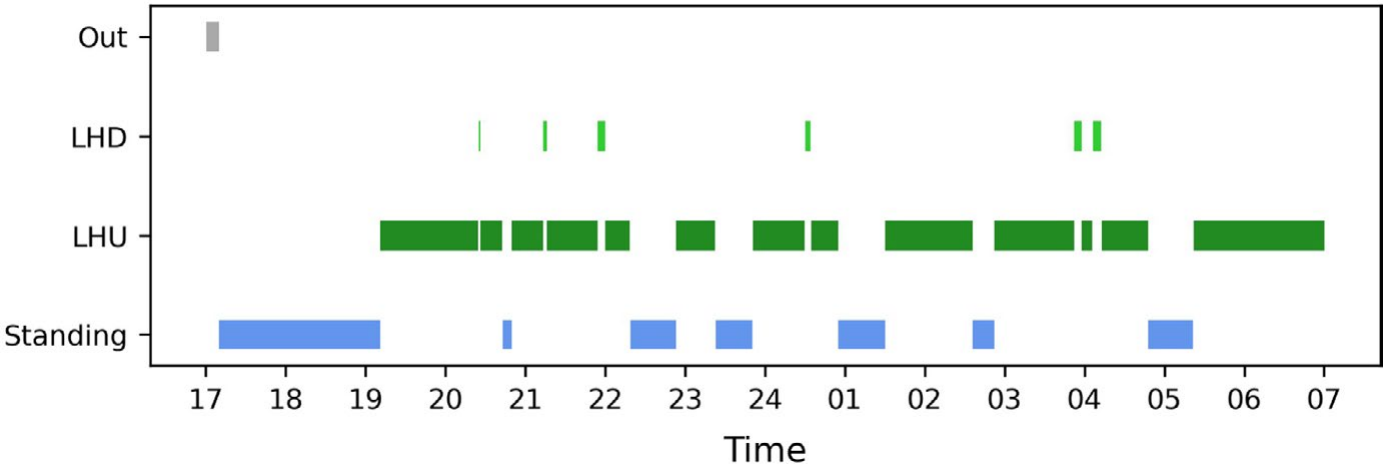
Example of one night of one common eland with the plotted phases of the three behavioral states of the total system given by BOVIDS to look for quality criteria QC 4

If QC 1–QC 3 are satisfactorily met, BOVIDS can be used to generate a final prediction of the unlabeled videos. Of course, QC 1 should be applied to unlabeled videos as well as it is a good indicator whether the object detector works well on a specific video. But even if the object detector detects an object quite frequently, it might happen that BOVIDS provides poor quality on a specific night if there are problems in the original data: for instance, individuals could be heavily truncated on a specific night. In those cases, it is reasonable to assume that the activity budget of the individual looks significantly different as in other videos which can be checked rather easily visually by searching for such outliers (QC 4). To this end, a short graphical representation of the activity budget in a video is generated by BOVIDS (see Figure 5) which can be used to identify those outliers. If the graphical representation of a night is conspicuous, one can check the original data on a sample basis to investigate BOVIDS’ performance.

##### DP 3: Data presentation

**FIGURE 6:**
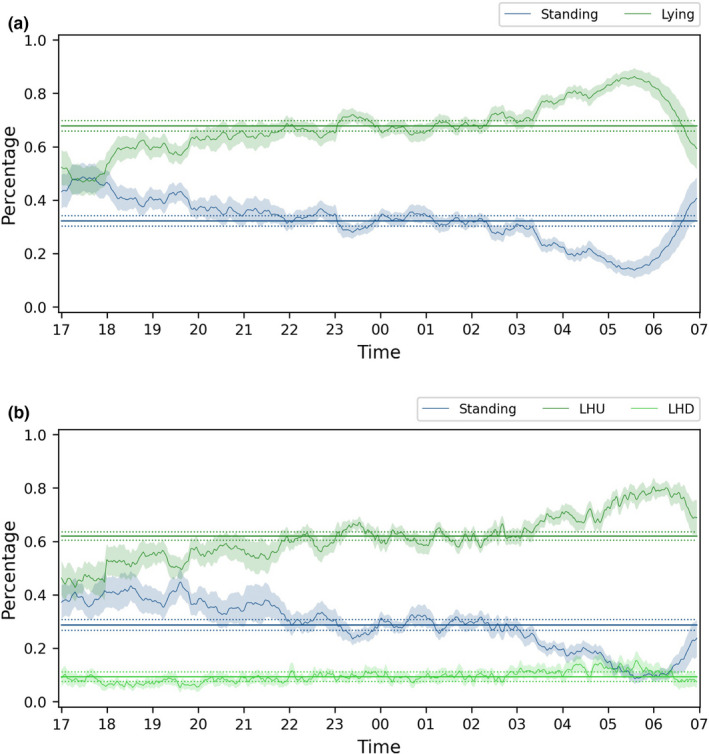
Timeline containing the percentage of all behavioral states and their means over all nights of all analyzed individuals of common elands. The visualization is smoothed by a rolling average over 3 min. (a) is the binary classification and contains 822 nights of 25 individuals, and (b) is the total classification containing 589 nights of 16 individuals

BOVIDS provides further functionalities to present the produced data elegantly which will be briefly described in this section and shown in more detail with the data of the case study in the results’ section. Next to the graphical representation (see QC 4) of each night, BOVIDS produces a document that contains an overview of the most important statistical key quantities, for instance, the percentages of the single behaviors in the activity budget. Finally, BOVIDS can be used to generate an overview about an individual's behavior over all evaluated nights or even about a species’ average behavior over all nights of all individuals. Furthermore, first graphical representations of the nightly activity are given as can be seen in Figure 6.

## 3 RESULTS

### 3.1 BOVIDS' performance in the case study

This section is devoted to reporting the validity of postprocessing rules and the quality criteria QC 1–QC 4 in the case study. Subsequently, in the next section, the nocturnal behavior of the common elands is presented.

A set of postprocessing rules can be considered as valid if the systematic error induced by these rules is negligible for the quantities of interest. In the dataset at hand and in both classification tasks, the accuracy of the postprocessed data ranges from 99.6% to 100% and even the f‐score of all activity classes lies constantly over 99.2%. Accordingly, the percentage per night per individual of all behavioral classes under both classification tasks are approximated up to an error of 0.02% in the worst case. Moreover, the average median phase length per individual is overshot by 21s of 1796s (Standing), 34s of 1375s (LHU) and 24s of 322s (LHD) in the total classification task while those values are 130s of 1834s (Standing) and 239s of 4226s (Lying) under binary classification. The number of phases per activity class is underestimated, more precisely, the mean deviation over all individuals is −0.29 of 8.2 (Standing), −1.02 of 23.0 (LHU), and −0.67 of 14.6 (LHD) in the total classification task while it is −1.4 of 8.9 (Standing) and −0.9 of 8.5 (Lying) in the binary classification system.

To analyze the quality criteria, the predictions of BOVIDS are compared to the manually annotated and postprocessed nights. All nights in which individuals were at least 20% of the time Out, either by BOVIDS’ prediction, or, if manually annotated by the humans’ prediction, were dismissed as such nights do not yield good evidence on the individual's activity budget. The results of all quality criteria are presented in this section.

On the analysis of the accuracy (QC 2) of BOVIDS’ prediction with respect to the manually annotated postprocessed data, the following results are found. The median accuracy per night lies at 99.4% with a 0.25‐quantile of 99.1% and a 0.75‐quantile of 99.4% in the total classification task. Furthermore, the median f‐scores turn out to be 99.6% (Standing), 99.5% (LHU), and 96.3% (LHD) with minima 94.4% (Standing), 95.4% (LHU), and 93.2% (LHD). In the binary classification task, the corresponding values read as follows. The median accuracy is 99.8% with a 0.25‐quantile of 99.4% and a 0.75‐quantile of 99.8% while the f‐scores are at least 93.1% (Standing) and 97.1% (Lying) with a median of 99.5% and 99.8%. Furthermore, the percentage of each behavioral class per individual is approximated up to at most 0.03% in both classification tasks. In the total classification system, the mean deviation in the number of phases is 0.34 of 7.9 (Standing), 0.53 of 22.0 (LHU), and 0.37 of 13.9 (LHD). The values in the binary classification task are 0.05 of 7.5 (Standing) and 0.03 of 7.6 (Lying). Finally, the median phase length per individual is underestimated by −22.6s of 1817.6s (Standing), by −117.0s of 1409.9s (LHU), and −1.8s of 345.6s (LHD) in the total classification task. In the binary classification system, those values turn out to be −2.87s of 1970.9s (Standing) and −14.7s of 4464.5s (Lying).

The next quality criteria to analyze is the number, length, and type of misclassified sequences (QC3). In the total classification task, we find, overall, 179 misclassified sequences in 62 nights (thus, on average, 2.9 sequences per night). Out of 179 sequences, 49 sequences are misclassifications between a real behavior and being Out and in 65 cases, BOVIDS predicted LHD while the actual behavior was LHU. The remaining 65 sequences were mostly short confusions between Standing and LHU. In contrast, in the binary classification task, there are 181 misclassified sequences in 170 nights (on average 1.1 sequences per night) out of which 78 are confusions between a behavioral class and Out, in 78 cases, BOVIDS predicts Standing while the human label is Lying and in 27 cases vice versa. Furthermore, out of the 181 sequences, 46 misclassifications are sequences of length at most 1 min and 47 additional misclassifications are below 5 min.

Quality criteria QC 1 and QC 4 are with respect to all predicted nights. Hereby, QC 1 checks the performance of the object detector. The detection density per individual ranges from 87.2% to 100% while its median turns out to be 99.8% with a 0.25‐quantile of 97.5% and a 0.75‐quantile of 100%. To analyze the last quality criteria (QC 4), namely, to look for apparent outliers, BOVIDS creates one plot per predicted night (for the binary and for the total classification task, respectively) representing the timely course of the behavioral phases (see Figure 5). There are few apparent outliers on data which were not manually labeled, and the automatic annotation was checked randomly. In most cases, it was found that BOVIDS’ prediction is correct even if it seemed to be suspicious. The observed misclassifications during this step fit exactly into the description of the errors in QC 3 and the frequency is comparable.

### 3.2 The nocturnal behavior of common elands

**FIGURE 7:**
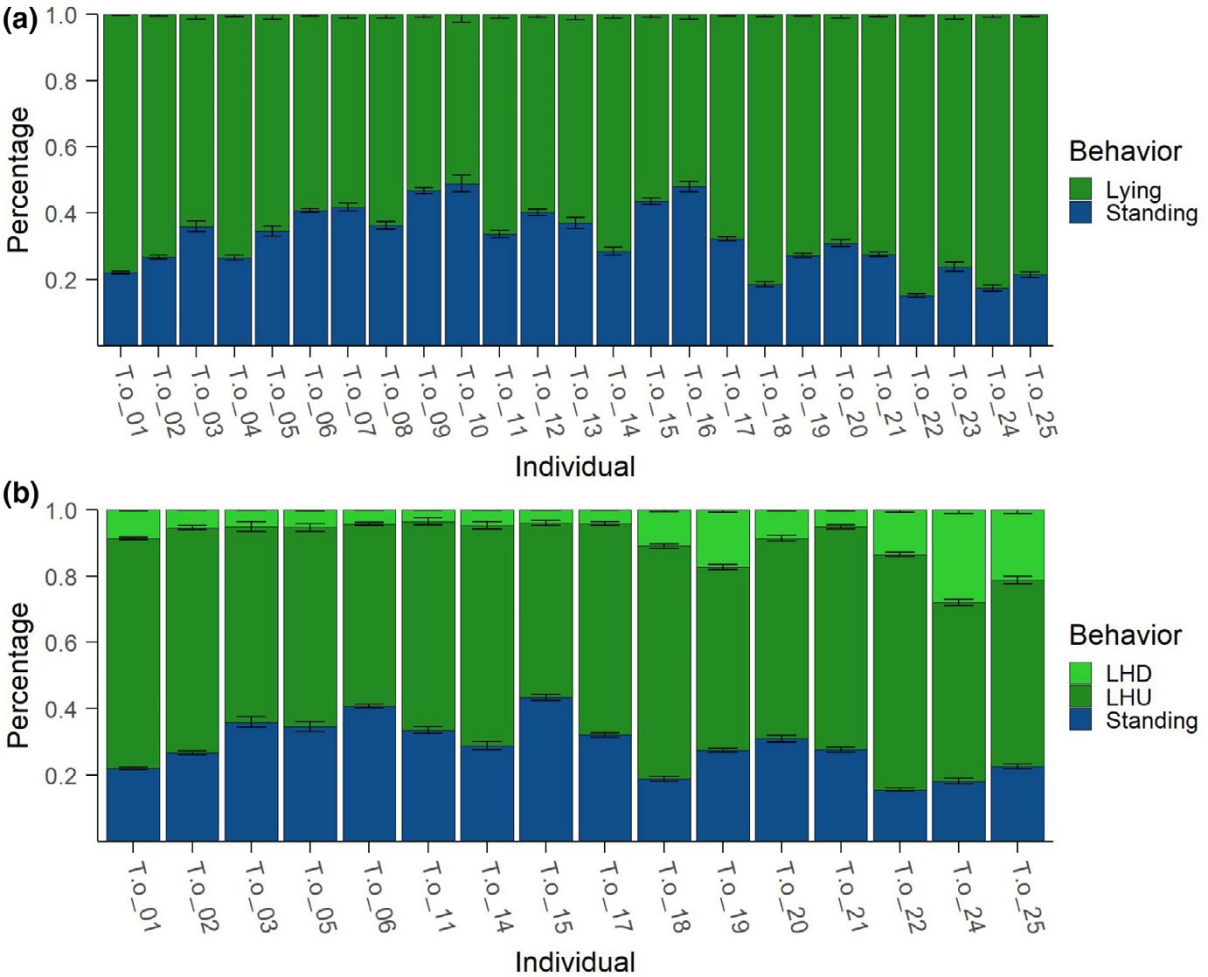
Activity budgets of all analyzed common elands: (a) is the binary classification with 822 nights of 25 individuals, and (b) is the total classification with 589 nights of 16 individuals. *T*.*oryx_01* to *T*.*oryx_05* are male adult individuals and *T*.*oryx_06* to *T*.*oryx_17* are female adult individuals, while *T*.*oryx_18* to *T*.*oryx_21* are subadults and *T*.*oryx_22* to *T*.*oryx_25* are young individuals

The data presentation tools of BOVIDS give a first visual result regarding the relative distribution of the behavioral states, their means over all nights, and the rhythm of phases of behavioral states (see Figure 6). The underlying data are normalized to the behavioral states excluding Out. The optically conjectured increase of Lying over the night between 19:00 and 06:00 in the binary classification task is confirmed by a linear regression (*R* ^2^ = .799 and *p* < .0001). In addition to the visual representation, BOVIDS’ output consists of tables, including a summary table for every individual containing relevant statistical key values as well as a list of the number of phases, durations, and the percentage of behaviors per night. This significantly facilitates the creation of an activity budget (see Figure 7) and provides a first insight into the nocturnal behavior of common elands. The graphical representation yields to the conjecture that there might be differences in the total duration of the behaviors per night between certain groups of individuals. Those differences are analyzed rigorously in the following.

**FIGURE 8:**
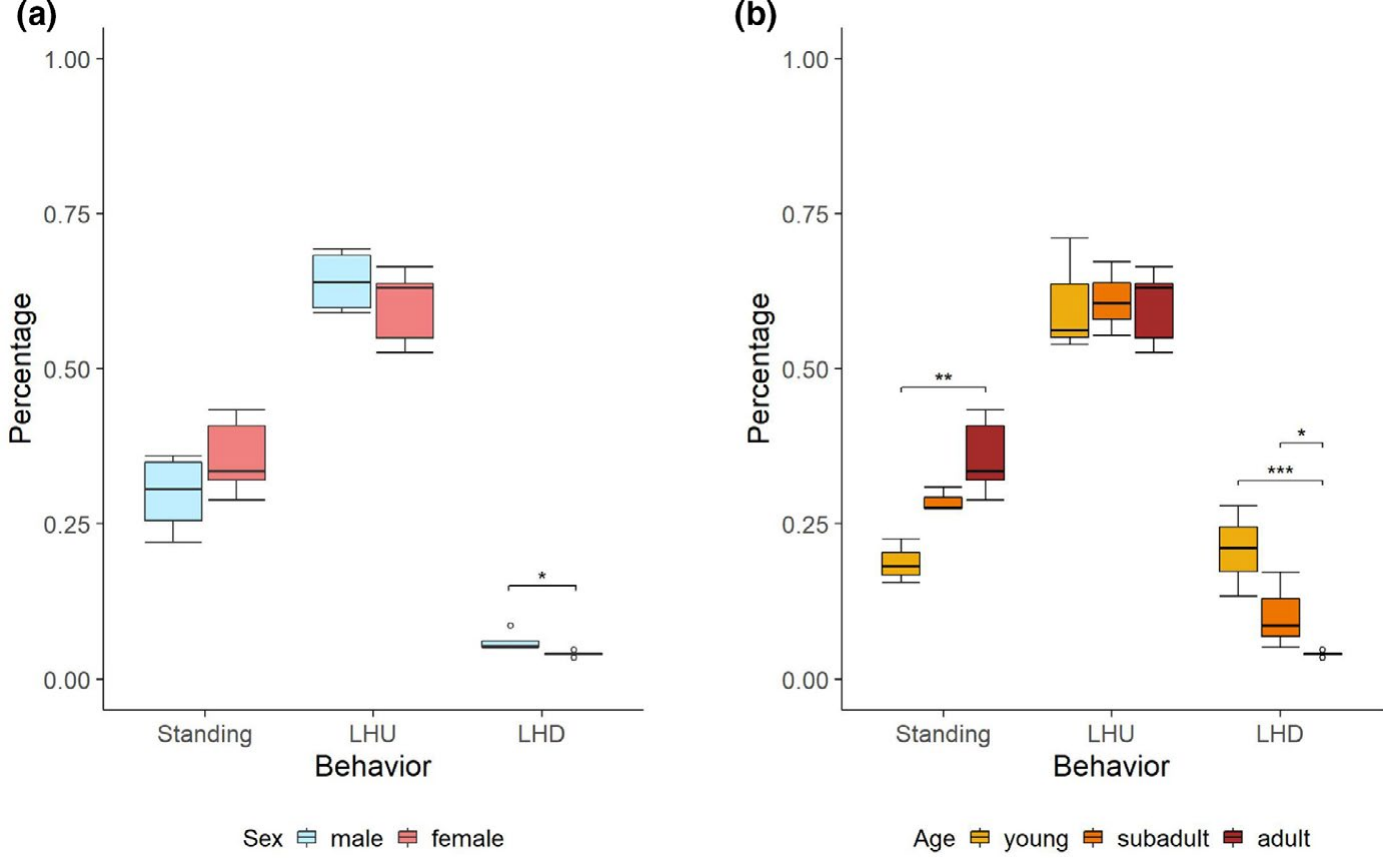
Comparison with respect to the total duration of each behavior per night in the total system. (a) Sex comparison (with *n* = 9 individuals with 328 nights, consisting of 4 males with 151 nights and 5 females with 177 nights) in which significant differences in LHD (*p* = .0281) arise. (b) Age comparison with (*n* = 11 individuals with 411 nights, consisting of 3 young individuals with 118, 3 subadults with 116 and 5 adults with 177 nights) that yields significant differences in Standing (p_young‐adult = .0038) and LHD (p_young‐adult = .0009; p_subadult‐adult = .0136)

The data with respect to Standing and LHU can be assumed to be normally distributed (p_Standing = 0.9524 and p_LHU = 0.2715) while the total duration per night of LHD deviates significantly from normality (p_LHD = 0.0015) and is transformed. First, adult male and adult female individuals are compared to investigate sex differences. Afterwards, age‐specific analyses’ will be conducted within the group of female individuals as there is only one nonadult male individual in the sample. To investigate the differences based on sex and to account for possible influences by the housing conditions, a two‐factor ANOVA is conducted with the factors keeping zoo and sex between the adult animals for each behavior of the total classification system (*n* = 9 individuals with 328 nights consisting of 4 males with 151 nights and 5 females with 177 nights). The holding zoo can be withdrawn as a significant factor (*p* > .37), but the sex has a significant influence on LHD (*p* = .0281), whereby the males’ values exceed the females’, see Figure 8(a). Finally, a two‐factor ANOVA with factors keeping zoo and age within all female individuals in the total classification system (n = 11 individuals with 411 nights consisting of 3 young with 118, 3 subadults with 116 and 5 adults with 177 nights) is conducted. Again, the holding zoo can be withdrawn as a factor (*p* > .58), but the age influences the total duration of Standing (p_young‐adult = 0.0038) and LHD (p_young‐adult = 0.0009; p_subadult‐adult = 0.0136) significantly as a corresponding post hoc analysis verifies. Hereby, nonadult individuals spend more time on LHD than adult ones, whereby adult ones spend more time Standing, see Figure 8(b). While the age comparison could only be carried out for female individuals, it is an advantageous circumstance that one individual could be recorded once as the subadult male individual (*T*.*oryx_18*) and moved during the observation phase to a different zoo in which it was observed as an adult male (*T*.*oryx_01*). This allows for a direct comparison of the behavior between the subadult and adult age of this individual as the husbandry conditions in the zoos studied were already considered negligible. An unpaired t‐test shows that the total amount of Standing (*p* < .0001) and LHD (*p* = .0001) differs significantly between the two observation periods of this individual, confirming the previously found results in differences due to age.

**FIGURE 9:**
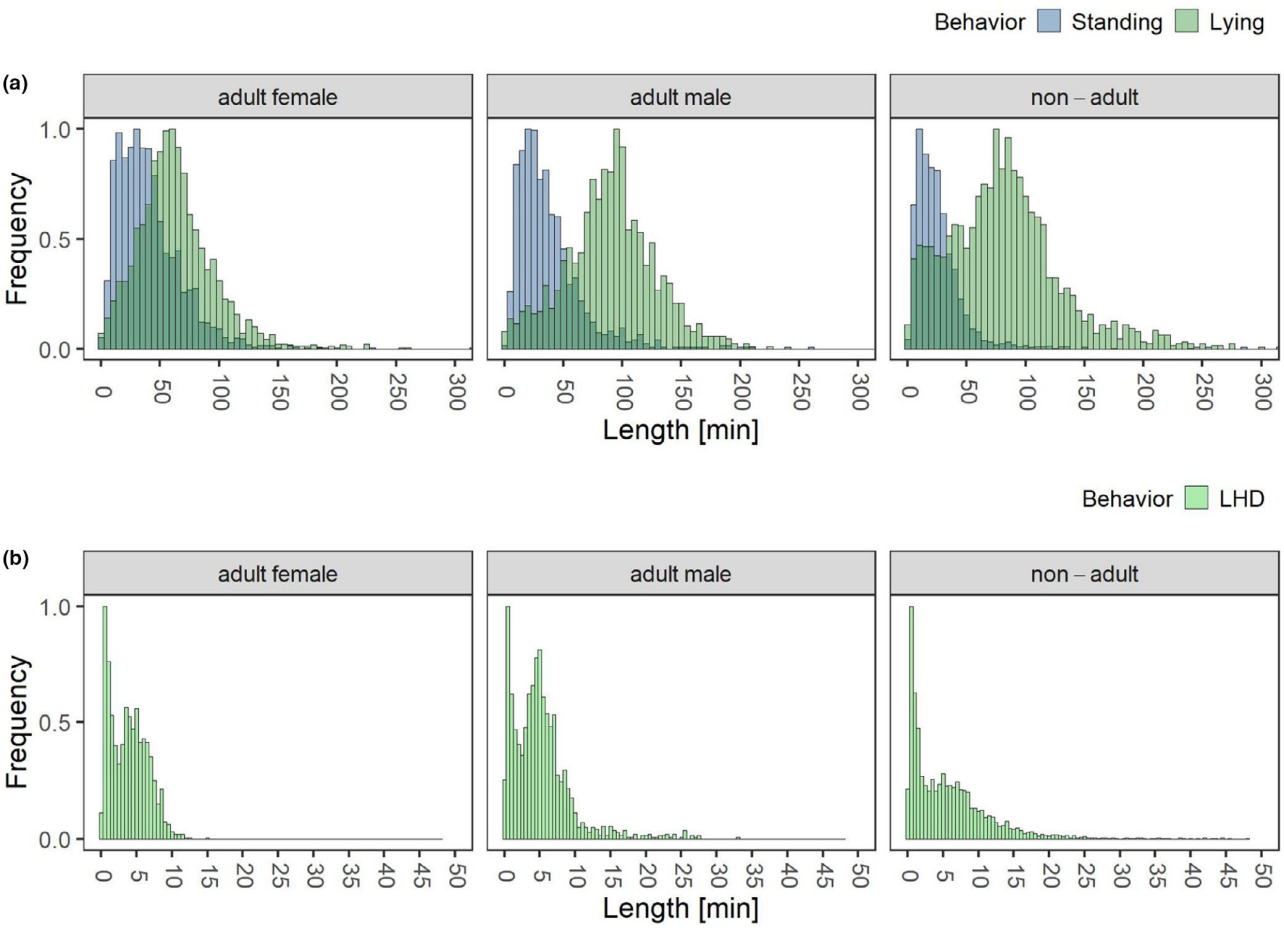
(a) For all 25 common elands, the distribution of the length of phases is in minutes of Standing and Lying from the binary classification task plotted and the animals are classified as adult male (*n* = 5 individuals with 179 nights), adult female (*n* = 12 individuals with 360 nights) and nonadult animals (*n* = 8 with 280 nights). (b) Only the 16 common elands evaluated by the total classification system are used. The length of phases in minutes of LHD are plotted and the animals are classified as adult male (*n* = 4 individuals with 151 nights), adult female (*n* = 5 individuals with 177 nights), and nonadult animals (*n* = 7 individuals with 261 nights)

A second variable of interest is the length of each behavioral phase. Regarding this quantity, the binary classification system (Standing and Lying) was used for the analysis as well as the duration of LHD from the total classification system as one Lying phase might be interrupted by several events of LHD. A Wilcoxon test reveals that there are significant differences (*p* = .0003) in the median length of phases per individual within Lying between males and females (*n* = 17 individuals with 539, consisting of 5 males with 179 nights and 12 females with 360 nights). For this reason, these two groups were analyzed separately. Within the females (*n* = 19 individuals with 613 nights, consisting of 4 young with 137 nights, 3 subadults with 116 and 12 adults with 360 nights), a post hoc analysis shows significant differences in the median duration of the Standing phases between young and adult individuals (p_Standing = 0.0033) and no significant differences between young and subadult animals (p_Standing = 0.1143, p_Lying = 0.629). Therefore, a detailed analysis is made after splitting into three categories, adult male, adult female, and nonadult (young and subadult) individuals. Figure 9 visualizes the distribution of the phase length regarding these categories. In median, the adult males exhibit the longest Lying phases with 89.6 min, followed by the nonadult animals (78.5 min) while the females show, with 59.3 min, the shortest Lying phases. While this is also true for the first and third quartile, the longest Lying event is achieved by the nonadults with 369.7 min. Within Standing, nonadult individuals seem to show a shorter median phase length (21.2 min) than adults (35.5 female, 30.8 male). With respect to phases of LHD, adult male individuals and nonadult individuals show, with a median value of 4.6 min and, respectively, 4.4 min a slightly longer duration than adult females with a median of 3.7 min. Nevertheless, the longest observed phase of LHD was by nonadult individuals (47.8 min) followed by the male adults (32.9 min) and the female adults (14.8 min).

**FIGURE 10:**
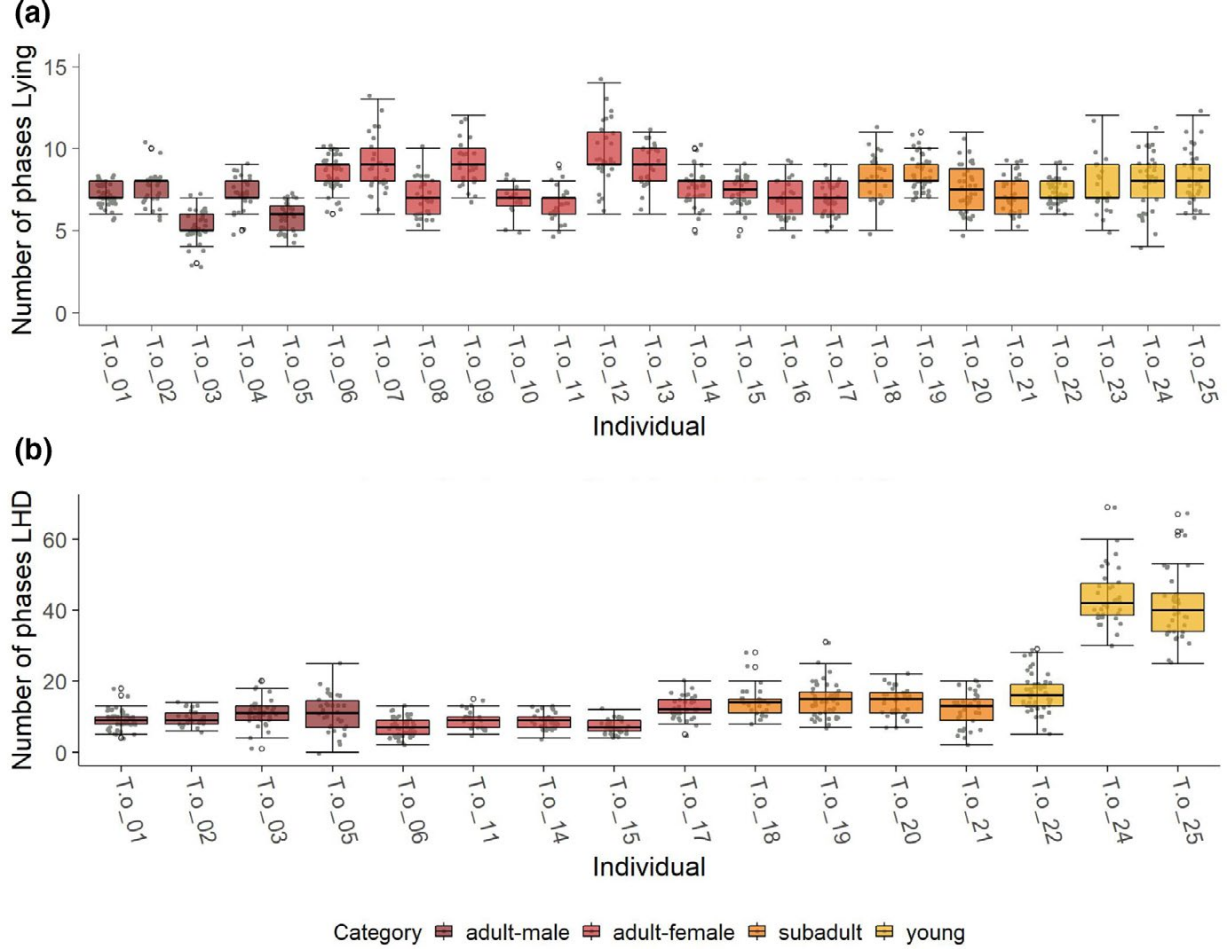
Number of phases for every individual marked are the groups, adult male, adult female, subadult, and young for (a) lying and (b) LHD

Beside the length of the phases, the number of phases per night is also an interesting parameter. Figure 10 visualizes the number of Lying phases (binary classification system) as well as the number of LHD phases (total classification system). Note that the number of Standing phases equals the number of Lying phases ±1. The above illustration highlights the different age categories of young, subadults, and adults, with sex being distinguished in the adult category. The phases in Lying (see Figure 10(a)) appear to be constant across individuals and differences between sex and age groups are not evident. The situation is different when it comes to LHD, where the young animals have a significantly higher number of phases than the adults. The subadults tend to have slightly more LHD phases than the adults, but they are already closer to the values of the adults than to those of the young.

## 4 DISCUSSION

### 4.1 BOVIDS

#### 4.1.1 Performance in the case study

In this section, the validity of the postprocessing rules as well as the four quality criteria are discussed. As can be seen in section *BOVIDS’ performance in the case study*, only very few activity phases are dismissed on manually annotated nights when the selected postprocessing rules are applied. Furthermore, both the accuracy and the f‐scores are close to 100%, so that overall, the set of postprocessing rules seems to be valid from a computer science point of view. Furthermore, the percentage of each behavioral class is very well approximated in both classification tasks, so that no mentionable errors occur. Not very surprisingly, the postprocessed data contains few phases less and slightly longer median phase lengths as very short events are dismissed, so the postprocessing rules imply almost no bias in the real data. These values are of course a bit higher in the binary classification task, since longer phases up to five minutes are not considered. But firstly, even this choice does not imply much bias in the data, and secondly, the few short events of Standing and Lying do not significantly affect the animals’ rhythms. Of course, neglecting the short events also increases the median phase length. However, this happens only very moderately, by a factor of between 5.6% (Standing) and 7.5% (LHD). It will be seen later that the methodological error will underestimate those quantities with respect to the postprocessed data slightly. Therefore, the errors partly account for each other.

The object detector seems to work very well (QC 1) as the median object detection density is very high. On nights with a lower detection density, the video material was checked manually, and it can be observed that the individuals were mostly Out if the object detector did not find them, or only small parts are visible at the border of the video recording.

Subsequently, quality criteria QC 2 and QC 3 are discussed. Since the number of phases per activity class and the phase length analysis refer to Standing and Lying from the binary classification task as well as LHD from the total classification task, the discussion focuses on the reliability of these quantities. Overall, the accuracy and the f‐score of BOVIDS’ prediction are very high for machine learning‐based predictions. Recent studies on comparable hard data, such as that of Porto et al. (2013) on the discrimination of Standing and Lying behavior on video recordings of cows in stables, achieve an average accuracy of 92%. Our accuracies of 99.8% in the binary classification task and 99.4% in the total classification task clearly exceed this value. Furthermore, even the median f‐score of the highly underrepresented class LHD is, with 96.4%, astonishingly high for a deep learning system. These values directly show that the percentage of each behavioral class is predicted very accurately and that there is no methodological bias in the expected activity budget.

Moreover, video action classifiers tend, normally, to so‐called classification flickering, thus very short phases of misclassifications which do not really influence the accuracy and the f‐score of the prediction system but have huge influence on the number of phases per activity. The postprocessing rules are meant to take care of this effect (Hahn‐Klimroth et al., 2021). The results show that BOVIDS succeeds in underestimating or overestimating the number of phases per activity class only very slightly on average. More precisely, the number of LHD phases is overestimated by 2.7% on average and the number of Standing and Lying phases is only overestimated by less than 1%. The median phase length is approximated very accurately as well, as it is only underestimated by at most 0.5% on average. It can be noted that even in enclosures containing two different individuals, BOVIDS’ prediction does not become significantly worse. This has two reasons: First, and most importantly, the used object detector seems to be able to discriminate between two individuals very accurately. Secondly, the action classifier seems to be very robust against truncation effects when, for example, the bounding boxes of the two animals overlap.

In summary, the activity budget per night is predicted without any bias, as expected, while the median phase length per activity class is overestimated due to postprocessing rules by a moderate factor of no more than 7.0%. Thus, the automatic prediction is very precise with respect to the postprocessed data. Furthermore, the overall accurate description of the three poses Standing, LHU, and LHD by BOVIDS can be seen in connection with the types of misclassifications occurring on the testing data. All misclassifications between Out and a real activity class are due to heavy truncation or occluding effects in which a human annotator might see hooves or small parts of the animal and is able to safely infer the behavior, but a machine cannot. In this case, it is favorable if the object detector does not find the animal in the first place. Furthermore, almost all misclassifications between LHU and LHD can be explained by the fact that common elands show, from time to time, a grooming behavior at their hind leg which is, on a single image, hard to distinguish from LHD. Such errors need to, of course, be considered and analyzed, but do not seem to be fixable by more training data or fine‐tuning the networks if the input data format does not change significantly. As mentioned earlier, the median phase length as well as the median number of phases per night are only slightly overestimated. In the binary classification task, there are some short misclassifications with respect to the postprocessed data less than five minutes in length. These errors are just delayed transitions between the behavioral states due to, for instance, the applied rolling average during postprocessing. Therefore, these misclassifications neither influence the number of phases of Standing and Lying nor the animal's rhythms, but only slightly change the total duration of a specific phase. Finally, there are few misclassifications that are, probably, unavoidable in a deep learning classification task. Of course, accuracy can, in principle, always be improved by additional rounds of example mining and fine‐tuning the action classifiers, but it is questionable whether an even higher median accuracy of 99.4% can be reached on a three‐class classification task.

A natural question, of course, is how well the findings from the test series can be generalized to unseen data of the same enclosures. Recall that the action classifiers are, in the end, trained on a random collection of images over the whole observation time due to offline hard example mining. Therefore, the testing set can be considered an almost random sample which includes a few more difficult images as expected on a random balanced sample. Thus, the analysis of the performance on the manually annotated nights (the testing set) yields a very good approximation of the overall performance. This claim is also supported by the analysis of QC 4. The type and frequency of errors on randomly selected, nonmanually annotated nights were found to be comparable to those in the test set.

Finally, even if BOVIDS makes a small number of mistakes that would not occur if a trained observer manually annotated the data, the very large dataset overcompensates those few errors. Another approach to generating a large dataset is to have different, probably untrained, human observers annotate a comparable number of nights. Apart from the much higher cost, it is supposed that the interobserver reliability might be worse than the reliability of BOVIDS. Overall, our findings show that BOVIDS performs very accurately in the case study and its predictions can be safely used to generate a large amount of annotated data, which would not have been easily possible without automation.

#### 4.1.2 Challenges and limitations

As for any deep learning‐based classifier, there are various challenges to overcome during fine‐tuning the underlying model. Even after extensive fine‐tuning, there will be cases in which the system fails. While the last paragraph already discussed that small errors are overcompensated by evaluation of much data, this section is devoted to exploring typical misclassifications that arise if BOVIDS is used.

A major challenge is given by highly truncated sequences of video material. In many applications, it is not possible to install cameras in a way that allows recording of every edge of the enclosure. This can cause misclassifications during action‐classification. Indeed, if only small parts of an animal can be seen, like only its hoofs or its head, and the object detector draws a decent bounding box, it is even for trained humans hard or even impossible to classify the behavior. To overcome this issue, it is possible to classify bounding boxes that are close to the image's border by a deterministic rule. A natural choice might be Out but in special cases one might use information about the recorded enclosure to infer the behavior in the truncated area. In‐depth observation of own data is necessary to identify those regions of the enclosure in which severe truncation effects might occur and to define proper rules on how to deal with them.

Another challenge arises if the animal is not present in a sequence of images. It is possible that an object in the enclosure like a trough might be falsely classified as an animal in this case. This issue can be addressed by more training steps of the object detector or by increasing the so‐called minimum confidence score: an object detector does not only suggest a bounding box and a class label but also returns a confidence score between zero and one. If a threshold of this value is defined near one, misclassifications are expected to be very rare, but the bounding boxes of animals are also more easily discarded. Finding a good threshold depends highly on the application and should, therefore, be tested.

A third type of errors might occur in enclosures in which multiple individuals are stalled together as the object detector might swap the individual's labels. In this case, short sequences of the proposed behavior can be false because the wrong individual is observed. There is no direct way to overcome this issue. In the case study, the object detector was tested excessively and worked very decently. But it is crucial to test the object detector's performance in the described fashion (see OD 3b). In future, implementations, one could extend BOVIDS to track bounding boxes from frame to frame. But on the technical side, the changes between consecutive frames might be too severe on recordings with 1fps to apply classical tracking methods. One possibility to deal with this problem would be to increase the recording's quality. This might give a second improvement. For instance, one could record with a much higher framerate that allows to use modern deep learning techniques like recurrent neural networks to capture the temporal dimension of the behavioral states more precisely. This comes with two challenges that may not be forgotten. First, it would require significantly more memory space. Second, it would also increase the computational cost. The current implementation predicts one hour of video material in approximately 5 min on mediocre hardware (RTX 2060 GPU) which would be exceeded significantly if more frames per second would need to be evaluated. If many video files need to be predicted in large‐scale studies, this might be a limiting factor. It is moreover to emphasize that under the described classification tasks the accuracy achieved by BOVIDS is highly satisfactory and it is unlikely that it can be much further improved. Nevertheless, techniques that use more temporal information might be able to capture short phases of certain behaviors more reliably. Behaviors that cannot be identified on a single image, or, more precisely, on four consecutive frames, cannot be detected in the current version. In the case study, grooming events at the hind legs (LHU) were sometimes predicted as LHD because the poses are close to each other. While normally misclassifications can be reduced by more rounds of offline hard example mining, it is presumably not possible to distinguish short grooming events and LHD within the given system. In the case study, these events were rare and therefore tolerable, but such analyses need to be conducted if the system should be applied to new data. During manual checking of samples, even trained humans were not able to reliably distinguish between those events and LHD on the given data. Of course, if the raw video material is used, this task is much easier, and one might hope to describe such events even more accurately using different architectures.

#### 4.1.3 Universality and future directions

A major strength of BOVIDS might be its adjustability to different settings. If the three positions Standing, LHU, and LHD need to be detected from video files, the system can be used on data of ungulates. Furthermore, in principle, any pose that is reliably detectable on single images can be predicted by the discussed deep learning framework. BOVIDS is tested extensively on the data of common elands and other African bovids stemming from various zoo enclosures. It is, therefore, reasonable to assume that, given sufficient training material, its performance is equally high under varying conditions. For instance, it is likely to perform well in the observation of various ungulates of different sizes from multiple continents in zoo enclosures or the analysis of livestock's behavior in stables. Since the present data are recorded in very different enclosures with partly high degrees of truncation and background noise, BOVIDS might perform well in outdoor enclosures as well if the camera installment is flawlessly possible in the sense that the whole outdoor enclosure can be recorded which would extend the set of research questions that can be tackled with this technique.

A further research direction would be the analysis of BOVIDS’ performance on data of larger groups of ungulates. While technically the detection of individuals works the same, it is clearly a much more difficult task to distinguish many individuals from each other than it is to identify two individuals reliably. It might be tempting to extend BOVIDS’ functionality in cases in which reliable distinguishing between different individuals is not possible. This might be due to the number of individuals and their optical similarity. For instance, if individual detection fails in large groups, one could implement a scan‐sampling method that allows to at least report an average behavior of all the individuals.

Moreover, the object detection phase can be used to identify different behavioral classes. If during a phase of Standing the bounding box's positions exhibit strong variability, this is a good indication for movement of the animal. Furthermore, it is possible to describe the individual's favorite positions within its enclosure and to keep track of the probability of the presence of the individuals at different spatial positions which can help to improve housing conditions in zoos. Both extensions suffer one technical challenge. Normally, one camera records an enclosure and, therefore, one can only work with a two‐dimensional projection of the actual positions. Depending on the camera positioning, movements into certain directions cannot be captured correctly. The same challenge applies to the description of the probability of the presence at spatial positions. If due to the camera's angle the bounding boxes are quite large in comparison to the whole image, such a description becomes meaningless. But overall, we believe that in many enclosures this approach can be implemented within the current deep learning system and can deliver more information on ungulate's behavior.

Furthermore, it is to discuss whether the iterative process used to create a reasonable training set could be improved. The degree of automation of the system at hand resembles more the one of a “machine‐assisted” evaluation of video material than the one of an autonomous deep learning system. Such iterative processes to obtain reliable machine learning models is extensively studied in a recent publication of Miao et al. (2021) at the example of camera‐trap images. The findings of the aforementioned publication as well as the findings of the current paper indicate that such a partly automated system reduces the time required by a researcher to evaluate data dramatically.

A similar question arises regarding the technical details of the training step of the action classifiers. To conquer data imbalance, the current contribution employs upsampling and downsampling techniques (Branco et al., 2016) and achieves good results. Nevertheless, it is tempting to try different training procedures to deal with the imbalance, as recently suggested by Liu et al. (2019).

Finally, it was already discussed that the deep learning architectures yolov4 and EfficientNet‐B3 are used because they are fast and show state‐of‐the‐art performance on testing sets. In principle, those architectures can be easily replaced if a novel approach performs even better. It is important to emphasize that the technical main contribution of BOVIDS is the sequential application of an object detector and a pair of action classifiers that capture the spatial and temporal dimension of the video data in the described fashion. The explicit implementation of these classifiers is independent from this approach and, therefore, it might be tempting to conduct comparative studies regarding the performance of different recent deep learning architectures within the proposed system.

### 4.2 The nocturnal behavior of common elands

A first finding is that independent from the factors age, sex, and keeping zoo, all individuals exhibit a higher percentage of Lying than Standing during the night. As the night progresses, the percentage of Lying increases significantly. This is in line to findings of similar studies on African elephants (*Loxodonta africana*), blue wildebeest (*Connochaetes taurinus*), or Arabian oryx (*Oryx leucoryx*), where the observed animals also show most of the sleeping behavior or inactivity in the second part of the night (Davimes et al., 2018; Gravett et al., 2017; Malungo et al., 2021).

When considering the LHD, it should be noted that this posture most likely corresponds to the typical REM (rapid eye movement) sleep posture. As mentioned in the ethogram section, a behavioral component to recognize REM sleep is the head being down due to postural atonia (Lima et al., 2005; Zepelin et al., 2005). In this study, we use this characteristically REM sleep posture to determine REM sleep. This approach is in line with the study by Zizkova et al. (2013) on common elands and the study by Ternman et al. (2014) on cows, which shows that REM sleep can be detected with sufficient certainty based on behavioral surveys. This procedure is also supported by a study on lesser mouse‐deer (*Tragulus kanchil*), which shows that REM sleep can be divided into two categories, one of which is the most common, where the head lies down most of the time, making this a valid indicator to recognize REM sleep in behavioral studies (Lyamin et al., 2021).

Sex has been found to have an influence on the total amount of LHD during the night. The REM sleep periods of adult females last slightly longer than those of adult males, a fact which is also known across multiple phylogenetic states, for birds and mammals (Cajochen et al., 2006; Rattenborg et al., 2017; Steinmeyer et al., 2010). However, other studies show that there are no sex differences when individuals are similar sized between the sexes, while dissimilar‐sized animals should have differences (Ruckstuhl & Kokko, 2002). In common elands, males are larger than females (Leslie, 2011; Myers et al., 2021), confirming the differences found between the sexes. In addition, Standing was found to increase with age. Interestingly, this finding is supported by the recording of a male individual at both subadult and adult age, which shows a significant increase in the total amount of Standing per night. Our results are in line with previous results on different mammals, as age is known to be an influencing factor for activity/rest cycles (Ruckstuhl & Neuhaus, 2009; Siegel, 2005; Steinmeyer et al., 2010). Moreover, age also influences REM sleep behavior in mammals and birds (Cajochen et al., 2006; Rattenborg et al., 2017; Ruckstuhl & Kokko, 2002; Steinmeyer et al., 2010). This effect was also observed in the common elands in this study, where the extent of LHD differs between the three age classes—young, subadults, and adults. A study on Giraffes (*Giraffa camelopardalis*) also shows that age and sex have an influence on the behavior Standing, while only age has an influence on REM sleep (Burger et al., 2021). The study by Burger et al. (2021) further reveals that housing conditions can be discarded as an influencing factor for both behaviors. These results correspond to the results in this study with common elands, where the keeping zoo and thus housing conditions can also be discarded as influencing factors. Of course, the factor housing condition consists of several factors such as, among others, enclosure size, and the presence or absence of other types of animals in the vicinity or lighting conditions. While the recorded data do not allow to evaluate each possibly influencing factor individually, our study reveals that the sum of those effects is negligible and can be discarded.

Besides the total amount of time during the night, the duration of the single phases is also of interest. Here, the males differ from the females within Lying, whereby males show longer Lying phases than females. This fits with the result that adult males have a higher amount of LHD. Also, the age has an influence on the lengths of the phases. The nonadult animals exhibit shorter periods of Standing and longer periods of Lying than the adult ones. This also matches with the results regarding the nocturnal activity budgets. Within LHD the number of phases vary between the different categories of individuals. The mean phase length of LHD in all adult common elands is 9.5 min on average, with females slightly below this at 8.8 min and males slightly above at 10.2 min. These phase lengths are consistent with those of male Arabian oryx (*Oryx leucoryx*), which have a mean phase length of 7 ± 2 min in the dark in winter, and 10.5 ± 1.5 min over the 24‐h cycle (Davimes et al., 2018).

Finally, the number of phases is an interesting key figure in behavioral analysis. Within Lying and Standing it is noticeable that almost all animals show a very similar number of phases. Here, of the 25 animals observed, 23 have a median between 7 and 9 phases per night with quite a little variation per individual. The other two animals are moderate outliers downward. In addition, the mean ranges between 6.6 and 9.1 within 22 individuals and within all individuals, the SEM is at most 0.36 indicating a constant behavior within the single individuals. This result suggests that certain rhythms are present and should be investigated in more detail in further analyses, because the course over the night also suggests certain rhythms. Within LHD, a different picture of the underlying distributions emerges. Here, the adult individuals show a lower proportion than the nonadult individuals, and within the nonadult individuals there are also strong differences between the young and the subadult individuals. Only a few exceptions are evident, which can be explained as follows. *T*.*oryx_22* is clearly different from the veined young and is closer to the values of the subadult individuals. However, *T*.*oryx_22* is also the oldest animal among the group of young ones. Furthermore, *T*.*oryx_17*, which is the oldest animal in the case study, has a higher median number of phases than the other adult animals, especially the female ones. Excluding these exceptions, young individuals have a median of 40–42 phases of LHD and subadults show 13–15 phases. In contrast, adult females have 7–9 phases of LHD and adult males 9–11 phases. This again indicates differences between the sexes and high similarities within each group of individuals. Again, it seems that certain rhythms are present depending on the sex and the age but being independent of the specific individual. This observation might be the starting point of a much more detailed analysis of rhythms in African ungulates’ behavior.

## CONFLICT OF INTEREST

The authors declare that the research was conducted in the absence of any commercial or financial relationships that could be construed as a potential conflict of interest.

## AUTHOR CONTRIBUTIONS

**Jennifer Gübert:** Conceptualization (lead); Data curation (lead); Formal analysis (lead); Methodology (supporting); Visualization (equal); Writing – original draft (equal). **Max Hahn‐Klimroth:** Formal analysis (supporting); Methodology (lead); Software (lead); Visualization (supporting); Writing – original draft (equal). **Paul W. Dierkes:** Funding acquisition (lead); Project administration (lead); Supervision (lead); Visualization (equal); Writing – original draft (supporting).

## Data Availability

The python code is available at GitHub: https://github.com/Klimroth/BOVIDS and on Zenodo (https://doi.org/10.5281/zenodo.6143896).

## APPENDIX A

### Overview data

A detailed overview about the used data is given in Table A1. Hereby, for every individual the categories age, sex, and the keeping zoo as well as the stabling conditions are contained. The exact age of the observed individuals ranges from one month to 16.5 years categorized as follows: “young” ranges from birth until the time of weaning with about 6 months, then the individuals become “subadult” until sexual maturity with about 2 years of age and after that they are listed as “adult.”

**TABLE A1:** The common elands observed in this study and their individual factors age (categorical: young, subadult and adult) and sex

Individual	Age	Sex	Keeping	Stabling	Nights	Duration (h)	Nights per hand	Pictures	Binary	Total
*T.oryx_01*	Adult	m	Zoo_1	Single	49	17–7	2	404	x	x
*T.oryx_02*	Adult	m	Zoo_4	Single	29	17–7	10	544	x	x
*T.oryx_03*	Adult	m	Zoo_3	Single	38	18–7	2	517	x	x
*T.oryx_04*	Adult	m	Zoo_5	Single	28	17–7	15	860	x	—
*T.oryx_05*	Adult	m	Zoo_2	Single	35	17–7	4	519	x	x
*T.oryx_06*	Adult	f	Zoo_1	Single	49	17–7	2	404	x	x
*T.oryx_07*	Adult	f	Zoo_4	Single	29	17–7	10	487	x	—
*T.oryx_08*	Adult	f	Zoo_4	Single	29	17–7	10	519	x	—
*T.oryx_09*	Adult	f	Zoo_4	Single	29	17–7	10	504	x	—
*T.oryx_10*	Adult	f	Zoo_4	Single	15	17–7	10	512	x	—
*T.oryx_11*	Adult	f	Zoo_3	Single	21	18–7	2	550	x	x
*T.oryx_12*	Adult	f	Zoo_5	Single	28	17–7	11	513	x	—
*T.oryx_13*	Adult	f	Zoo_5	Single	28	17–7	14	541	x	—
*T.oryx_14*	Adult	f	Zoo_2	Together	35	17–7	2	604	x	x
*T.oryx_15*	Adult	f	Zoo_2	Together	34	17–7	2	604	x	x
*T.oryx_16*	Adult	f	Zoo_4	Single	25	17–7	10	557	x	—
*T.oryx_17*	Adult	f	Zoo_3	Single	38	18–7	2	511	x	x
*T.oryx_18*	Subadult	m	Zoo_5	Together	27 (28)	17–7	17 (18)	502	x	x
*T.oryx_19*	Subadult	f	Zoo_1	Together	49	17–7	2	636	x	x
*T.oryx_20*	Subadult	f	Zoo_2	Single	34	17–7	4	519	x	x
*T.oryx_21*	Subadult	f	Zoo_2	Single	33	17–7	4	519	x	x
*T.oryx_22*	Young	f	Zoo_1	Together	49	17–7	2	636	x	x
*T.oryx_23*	Young	f	Zoo_5	Together	22 (28)	17–7	15 (18)	502	x	—
*T.oryx_24*	Young	f	Zoo_2	Together	35	17–7	2	604	x	x
*T.oryx_25*	Young	f	Zoo_2	Together	34	17–7	2	604	x	x

Note

Further, the housing zoo and the given stabling conditions (standing single or together), are contained. The duration gives the recording start and end time and the totally recorded number of nights as well as the manually annotated number of nights are listed, if nights had to be removed because of an object detection density score smaller than 80% the used number of nights are listed with the real number of nights in parentheses. Finally, the number of pictures describes the number of annotated images in the object detection training set after OHEM. Observe that *T.oryx_01* and *T.oryx_18* is the same individual recorded at different times after moving from one zoo to another. Also, it is marked if the individuals are evaluated with the total or binary classification system.

### Postprocessing rules

This section contains the post‐processing rules applied to BOVIDS’ prediction for both classification tasks. With respect to the total classification task, different sets of rules are applied for adult common elands and nonadult common elands, because nonadult individuals show shorter phases.

The order of the applied rolling average varies between the three sets of rules. A higher order reduces flickering but is likely to dismiss (very) short events. Therefore, the order of the rolling average was set to 3 in the total classification task for nonadult individuals, to 4 in the total classification task for adult individuals and to 5 in the binary classification task.

Regarding dismissing short phases, the quantity “minimum length” is introduced followed by a three‐character code. If this code is XYZ, this is meant to be read as follows. Suppose a phase of behavior Y lies in between a phase of behavior X and behavior Z, then the event will be dismissed (marked as X) if it consists of less time‐intervals than indicated by the minimum length of XYZ. In those codes, Standing is abbreviated to “A,” LHU to “L” and LHD to “S” in the total classification task. In the binary classification task, “A” means Standing and “L” means Lying. “O” stands for Out in both tasks. *X* is meant to be read as any combination YXZ where Y and Z do not equal X. The applied rules of dismissing short phases can be found in Table A2.

Regarding the special state Out, the post‐processing rules are a bit more elaborated. If flickering between Out and a real behavioral state occurs, this is very likely due to a failure of the object detector if an animal is occluded or truncated. Therefore, if a sequence of a specific behavioral state X (Standing, Lying, LHU or LHD) is interrupted by phases of Out, the Out phases are dismissed under the following conditions. First, each single phase of Out must be shorter than 27 time‐intervals (total) or 135 time‐intervals (binary). Second, the total percentage of X in the sequence needs to exceed 20%.

**TABLE A2:** Overview about the minimum length a specific behavioral phase needs to have in order not to be dismissed in the post‐processing step

Behavior code	Total adult	Total nonadult	Binary
SLS	3	2	—
SLA	3	3	—
ALS	3	3	—
ALA	6	6	45
OLA	6	6	45
OLS	6	6	—
ALO	6	6	45
SLO	6	6	—
SAS	25	6	—
SAL	25	6	—
LAS	25	6	—
LAL	25	6	45
LAO	25	6	45
OAL	25	6	45
OAS	25	6	—
SAO	25	6	—
ASA	9	9	—
ASL	6	6	—
LSA	6	6	—
LSL	2	2	—
LSO	9	9	—
OSL	9	9	—
ASO	9	9	—
OSA	9	9	—
*O*	9	9	45

Note

The value is to be read as time‐intervals where 1 time‐interval consists of 7 seconds. Standing is abbreviated to “A,” LHU to “L” and LHD to “S” in the total classification task. In the binary classification task, “A” means Standing and “L” means Lying. “O” stands for Out in both tasks.
